# Supplementary material for: Design, Synthesis and Herbicidal Activity of 1,2,4-Oxadiazole Compounds as Novel Light-Dependent Protochlorophyllide Oxidoreductase Inhibitors
Source: Molecules. 2025 Oct 3;30(19):3970. doi: 10.3390/molecules30193970 (PMC12526218; doi:10.3390/molecules30193970)
Supplement: Supplementary file 1 [file molecules-30-03970-s001.zip › molecules-3900356-supplementary.pdf]

Catalogue

NMR Spectra of All Products.. 1

Figure S21. Postemergence herbicidal activity of Compounds 5j, 5k, 5q, SUT (150 g ai/ha, DS) 30

Figure S22. Crop Safety Experiment... 30

NMR Spectra of All Products

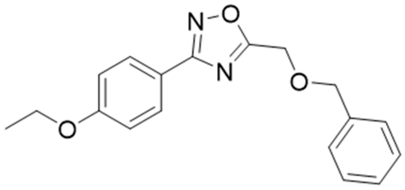

5-((benzyloxy)methyl)-3-(4-ethoxyphenyl)-1,2,4-oxadiazole (**5a**). Yield: 78%. White solid. m.p. 44−45 °C. ^{1}H NMR (500 MHz, CDCl3) δ 7.98 – 7.91 (m, 2H), 7.36 – 7.19 (m, 5H), 6.94 – 6.84 (m, 2H), 4.68 (s, 2H), 4.64 (s, 2H), 3.99 (q, J = 7.0 Hz, 2H), 1.35 (t, J = 7.0 Hz, 3H). ^{13}C NMR (500 MHz, CDCl3) δ 175.71, 168.19, 161.48, 136.58, 129.17, 128.64, 128.31, 128.21, 118.76, 114.77, 73.57, 63.64, 62.44, 14.75. HRMS (Dual ESI): Calcd for $C_{18}H_{19}N_2O_3$ $[M+H]^+$ 311.1317. Found 223.1398.

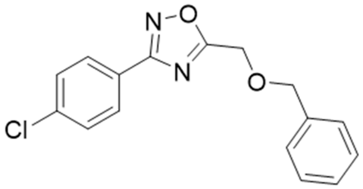

5-((benzyloxy)methyl)-3-(4-chlorophenyl)-1,2,4-oxadiazole (**5b**). Yield: 78%. Yellow oily. ^{1}H

NMR (500 MHz, $CDCl_3$) δ 8.01 – 7.85 (m, 2H), 7.42 – 7.18 (m, 7H), 4.69 (s, 2H), 4.64 (s, 2H) .^{13}C NMR (500 MHz, $CDCl_3$) δ 176.28, 167.67, 137.53, 136.47, 129.26, 128.87, 128.68, 128.38, 128.21, 125.04, 73.67, 62.38. HRMS (Dual ESI): Calcd for $C_{16}H_{14}ClN_2O_2$ $[M+H]^+$ 301.0666. Found 301.0747.

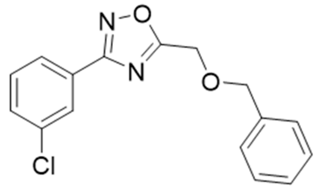

5-((benzyloxy)methyl)-3-(3-chlorophenyl)-1,2,4-oxadiazole (**5c**). Yield: 63%. Yellow oily. ^{1}H NMR (500 MHz, $CDCl_3$) δ 8.01 (t, J = 1.9 Hz, 1H), 7.89 (dt, *J* = 7.7, 1.4 Hz, 1H), 7.42 - 7.18 (m, 7H), 4.69 (s, 2H), 4.64 (s, 2H). ^{13}C NMR (500 MHz, CDCl3) δ 175.31, 166.41, 135.37, 133.94, 130.34, 129.18, 127.61, 127.32, 127.16, 126.59, 124.54, 72.61, 61.30. HRMS (Dual ESI): Calcd for $C_{16}H_{13}N_2O_2ClNa$ $[M+Na]^+$ 323.0563. Found 323.0566

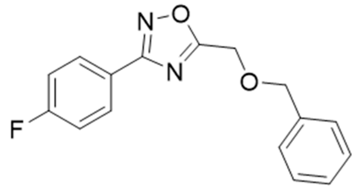

5-((benzyloxy)methyl)-3-(4-fluorophenyl)-1,2,4-oxadiazole (**5d**). Yield: 72%. Yellow oily. ^{1}H NMR (500 MHz, $CDCl_3$) δ 8.00 (dd, J = 8.7, 5.7 Hz, 2H), 7.43 - 7.17 (m, 5H), 7.06 (t, J = 8.7 Hz, 2H), 4.68 (s, 2H), 4.63 (s, 2H). ^{13}C NMR (500 MHz, $CDCl_3$) δ 176.19, 167.65, 165.69, 163.68, 136.51, 129.77, 129.70, 128.66, 128.36, 128.21, 122.81, 122.78, 116.20, 116.02, 73.64, 62.39. ^{19}F NMR (500 MHz, $CDCl_3$) δ -111.77 (d, *J* = 9.2 Hz). HRMS (Dual ESI): Calcd for $C_{16}H_{13}FN_2O_2$ $[M+H]^+$ 285.0961. Found 285.1033.

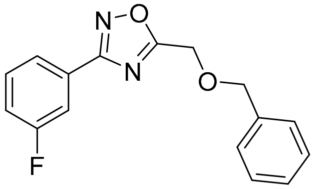

5-((benzyloxy)methyl)-3-(4-fluorophenyl)-1,2,4-oxadiazole (**5e**). Yield: 61%. White solid. m.p. 134.4−135.8 °C.^{1}H NMR (500 MHz, $CDCl_3$) δ 8.44 (t, *J* = 1.8 Hz, 1H), 8.04 (dt, *J* = 7.9, 1.4 Hz, 1H), 7.80 (dt, *J* = 8.0, 1.5 Hz, 1H), 7.44 – 7.25 (m, 5H), 7.17 (t, *J* = 7.9 Hz, 1H), 4.76 (s, 2H), 4.71 (s, 2H).^{13}C NMR (500 MHz, $CDCl_3$) δ 176.38, 167.14, 140.27, 136.49, 136.32, 130.58, 128.70, 128.46, 128.39, 128.23, 126.65, 94.50, 77.43, 73.70, 62.42. ^{19}F NMR (500 MHz, $CDCl_3$) δ -108.20. HRMS (Dual ESI): Calcd for $C_{16}H_{14}FN_2O_2$ $[M+H]^+$ 285.0961. Found 285.1036.

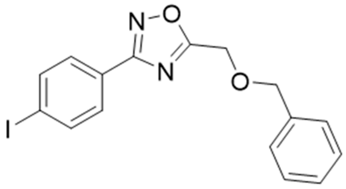

5-((benzyloxy)methyl)-3-(4-iodophenyl)-1,2,4-oxadiazole (**5f**). Yield: 71%. White solid. m.p. 39−40 °C.1H NMR (500 MHz, CDCl3) δ 7.75 (s, 4H), 7.37 – 7.20 (m, 5H), 4.71 (s, 2H), 4.66 (s, 2H).13C NMR (500 MHz, CDCl3) δ 176.28, 167.91, 138.18, 136.43, 129.04, 128.68, 128.22, 126.02, 98.14, 73.69, 62.38. HRMS (Dual ESI): Calcd for $C_{16}H_{14}IN_2O_2$ $[M+H]^+$ 393.0022. Found 393.0100.

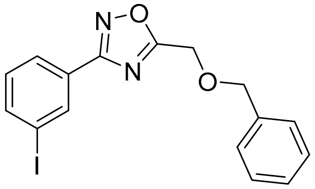

5-((benzyloxy)methyl)-3-(4-iodophenyl)-1,2,4-oxadiazole (**5g**). Yield: 82%. White solid. m.p. 134.4−135.8 °C.^{1}H NMR (500 MHz, $CDCl_3$) δ 7.80 (d, *J* = 7.8 Hz, 1H), 7.71 (d, *J* = 9.1 Hz, 1H), 7.41 – 7.18 (m, 6H), 7.11 (t, *J* = 8.4 Hz, 1H), 4.70 (s, 2H), 4.64 (s, 2H).^{13}C NMR (500 MHz, $CDCl_3$) δ 176.36, 167.62, 163.90, 161.94, 136.47, 130.68, 130.61, 128.67, 128.62, 128.55, 128.37, 128.22,

123.29, 123.26, 118.43, 118.27, 114.71, 114.52, 77.29, 73.67, 62.37. HRMS (Dual ESI): Calcd for $C_{16}H_{14}IN_2O_2$ $[M+H]^+$ 393.0022. Found 393.0099.

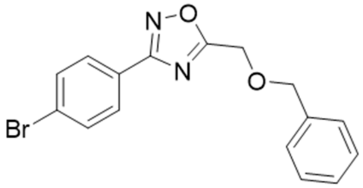

5-((benzyloxy)methyl)-3-(4-bromophenyl)-1,2,4-oxadiazole (**5h**). Yield: 65%. Yellow oily. ^1H NMR (500 MHz, $CDCl_3$) δ 7.92 - 7.82 (m, 2H), 7.57 - 7.48 (m, 2H), 7.36 - 7.19 (m, 5H), 4.69 (s, 2H), 4.64 (s, 2H). ^{13}C NMR (500 MHz, $CDCl_3$) δ 176.30, 167.76, 136.45, 132.22, 129.05, 128.69, 128.39, 128.23, 125.97, 125.48, 73.68, 62.38. HRMS (Dual ESI): Calcd for $C_{16}H_{14}BrN_2O_2$ $[M+H]^+$345.0160. Found 345.0232.

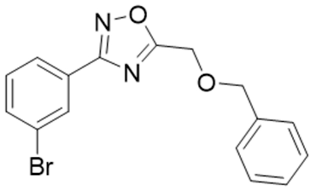

5-((benzyloxy)methyl)-3-(3-bromophenyl)-1,2,4-oxadiazole (**5i**). Yield: 61%. White solid. m.p. 31−32 °C. ^1H NMR (500 MHz, $CDCl_3$) δ 8.17 (s, 1H), 7.94 (d, *J* = 7.8 Hz, 1H), 7.53 (d, *J* = 7.0 Hz, 1H), 7.38 – 7.17 (m, 6H), 4.69 (s, 2H), 4.64 (s, 2H). ^{13}C NMR (500 MHz, $CDCl_3$) δ 176.39, 167.35, 136.44, 134.34, 130.54, 130.49, 128.69, 128.46, 128.40, 128.23, 126.05, 123.00, 73.69, 62.38. HRMS (Dual ESI): Calcd for $C_{16}H_{14}BrN_2O_2$ $[M+H]^+$ 345.0160. Found 345.0234.

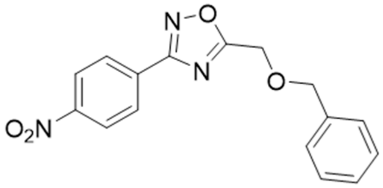

5-((benzyloxy)methyl)-3-(4-nitrophenyl)-1,2,4-oxadiazole (**5j**). Yield: 77%. White solid. m.p. 84−85 °C. ^1H NMR (500 MHz, $CDCl_3$) δ 8.29 - 8.14 (m, 4H), 7.41 - 7.19 (m, 5H), 4.74 (s, 2H), 4.67 (s, 2H). ^{13}C NMR (500 MHz, $CDCl_3$) δ 176.28, 167.67, 137.53, 136.47, 129.26, 128.87, 128.68,

128.38, 128.21, 125.04, 73.67, 62.38. HRMS (Dual ESI): Calcd for $C_{16}H_{14}N_3O_4$ $[M+H]^+$ 312.0906. Found 312.1007.

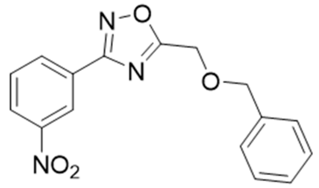

5-((benzyloxy)methyl)-3-(3-nitrophenyl)-1,2,4-oxadiazole (**5k**). Yield: 66%. White solid. m.p. 42−43 °C.^{1}H NMR (500 MHz, $CDCl_3$) δ 8.85 (t, J = 2.0 Hz, 1H), 8.38 – 8.20 (m, 2H), 7.59 (t, J = 8.0 Hz, 1H), 7.37 – 7.19 (m, 5H), 4.74 (s, 2H), 4.67 (s, 2H).^{13}C NMR (500 MHz, $CDCl_3$) δ 176.93, 166.88, 148.65, 136.36, 133.11, 130.13, 128.70, 128.43, 128.33, 128.23, 125.87, 122.64, 77.31, 73.79, 62.36. HRMS (Dual ESI): Calcd for $C_{16}H_{14}N_3O_4$ $[M+H]^+$312.0906. Found 312.0978.

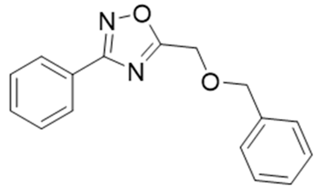

5-((benzyloxy)methyl)-3-phenyl-1,2,4-oxadiazole (**5l**). Yield: 85%. Yellow oily. ^{1}H NMR (500 MHz, $CDCl_3$) δ 8.09 – 7.95 (m, 2H), 7.48 – 7.16 (m, 8H), 4.68 (s, 2H), 4.63 (s, 2H).^{13}C NMR (500 MHz, $CDCl_3$) δ 174.97, 167.38, 135.48, 130.27, 127.83, 127.57, 127.24, 127.13, 126.48, 125.48, 72.52, 61.34,. HRMS (Dual ESI): Calcd for $C_{16}H_{15}N_2O_2$ $[M+H]^+$ 267.1055. Found 267.1127.

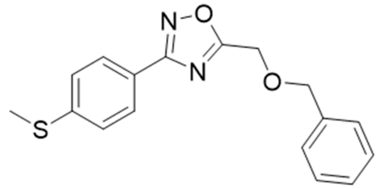

5-((benzyloxy)methyl)-3-(4-(methylthio)phenyl)-1,2,4-oxadiazole (**5m**). Yield: 61%. White solid. m.p. 44−45 °C.^{1}H NMR (500 MHz, $CDCl_3$) δ 7.97 - 7.86 (m, 2H), 7.37 - 7.19 (m, 7H), 4.70 (s, 2H), 4.65 (s, 2H), 2.44 (s, 3H).^{13}C NMR (500 MHz, $CDCl_3$) δ 175.94, 168.13, 143.18, 136.52, 128.66,

128.35, 128.22, 127.80, 125.87, 122.79, 73.62, 62.42, 15.08. HRMS (Dual ESI): Calcd for $C_{17}H_{17}N_2O_2S$ $[M+H]^+$ 313.0932. Found 313.1006.

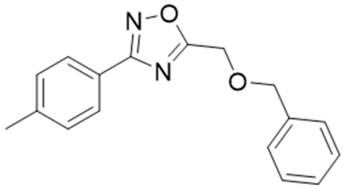

5-((benzyloxy)methyl)-3-(p-tolyl)-1,2,4-oxadiazole (**5n**). Yield: 83%. Yellow oily. ^1H NMR (500 MHz, $CDCl_3$) δ 7.94 - 7.82 (m, 2H), 7.34 - 7.11 (m, 7H), 4.66 (s, 2H), 4.61 (s, 2H), 2.29 (s, 3H). ^{13}C NMR (500 MHz, $CDCl_3$) δ 174.77, 167.33, 140.61, 135.45, 128.53, 127.55, 127.22, 127.13, 126.38, 122.59, 72.47, 61.33, 20.51. HRMS (Dual ESI): Calcd for $C_{17}H_{17}N_2O_2$ $[M+H]^+$ 281.1212. Found 281.1273.

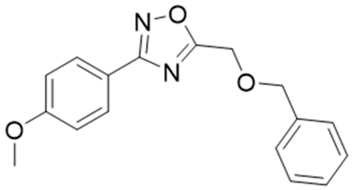

5-((benzyloxy)methyl)-3-(4-methoxyphenyl)-1,2,4-oxadiazole (**5o**). Yield: 70%. Yellow oily. ^1H NMR (500 MHz, $CDCl_3$) δ 8.02 - 7.89 (m, 2H), 7.37 - 7.19 (m, 5H), 6.96 - 6.86 (m, 2H), 4.69 (s, 2H), 4.65 (s, 2H), 3.78 (s, 3H). ^{13}C NMR (500 MHz, $CDCl_3$) δ 174.68, 167.10, 161.02, 135.51, 128.13, 127.59, 127.26, 127.17, 117.93, 113.25, 72.52, 61.38, 54.35. HRMS (Dual ESI): Calcd for $C_{17}H_{17}N_2O_3$ $[M+H]^+$ 297.1161. Found 297.1237.

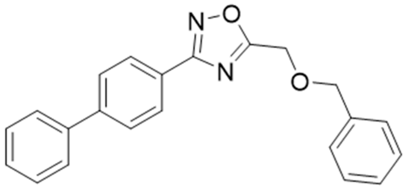

3-([1,1'-biphenyl]-4-yl)-5-((benzyloxy)methyl)-1,2,4-oxadiazole (**5p**). Yield: 82%. White solid. m.p. 81−82 °C. ^1H NMR (500 MHz, $CDCl_3$) δ 8.08 (d, J = 6.9 Hz, 2H), 7.62 (d, J = 8.0 Hz, 2H), 7.54 (d, J = 7.6 Hz, 2H), 7.46 – 7.17 (m, 8H), 4.70 (s, 2H), 4.65 (s, 2H). ^{13}C NMR (500 MHz, $CDCl_3$)

δ 176.08, 168.29, 144.12, 140.16, 136.56, 128.97, 128.69, 128.37, 128.25, 128.03, 127.59, 127.21, 125.39, 73.65, 62.47. HRMS (Dual ESI): Calcd for $C_{22}H_{19}N_2O_2$ $[M+H]^+$ 343.1368. Found 343.1464.

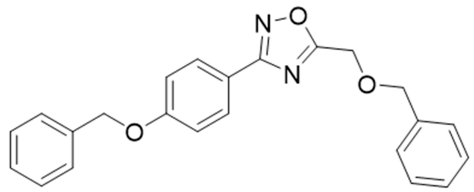

5-((benzyloxy)methyl)-3-(4-(benzyloxy)phenyl)-1,2,4-oxadiazole (**5q**). Yield: 64%. White solid. m.p. 134.4−135.8 °C.^{1}H NMR (500 MHz, $CDCl_3$) δ 8.07 - 7.99 (m, 2H), 7.49 - 7.25 (m, 10H), 7.09 - 7.01 (m, 2H), 5.10 (s, 2H), 4.76 (s, 2H), 4.71 (s, 2H).^{13}C NMR (500 MHz, $CDCl_3$) δ 175.78, 168.14, 161.26, 136.59, 136.46, 129.23, 128.72, 128.67, 128.34, 128.21, 127.55, 119.25, 115.21, 73.59, 70.11, 62.45. HRMS (Dual ESI): Calcd for $C_{23}H_{21}N_2O_3$ [M+H]+ 373.1474. Found 373.1548.

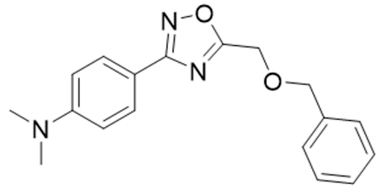

4-(5-((benzyloxy)methyl)-1,2,4-oxadiazol-3-yl)-*N,N*-dimethylaniline (**5r**). Yield: 64%. White solid. m.p. 67−68 °C.^{1}H NMR (500 MHz, $CDCl_3$) δ 7.94 - 7.81 (m, 2H), 7.44 - 7.19 (m, 5H), 6.71 - 6.58 (m, 2H), 4.67 (s, 2H), 4.63 (s, 2H), 2.94 (s, 6H).^{13}C NMR (500 MHz, $CDCl_3$) δ 175.24, 168.56, 152.28, 136.67, 133.42, 128.76, 128.63, 128.27, 128.23, 113.64, 111.69, 111.43, 73.52, 62.51, 40.16. HRMS (Dual ESI): Calcd for $C_{18}H_{20}N_3O_2$ $[M+H]^+$ 310.1477. Found 310.1561.

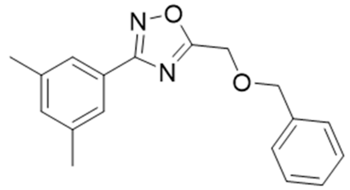

5-((benzyloxy)methyl)-3-(3,5-dimethylphenyl)-1,2,4-oxadiazole (**5s**). Yield: 72%. Yellow oily. ^{1}H NMR (500 MHz, $CDCl_3$) δ 7.68 - 7.60 (m, 2H), 7.36 - 7.19 (m, 5H), 7.05 (s, 1H), 4.70 (s, 2H), 4.64

(s, 2H), 2.29 (s, 6H).^{13}C NMR (500 MHz, $CDCl_3$) δ 175.86, 168.64, 138.62, 136.55, 133.07, 128.66, 128.33, 126.23, 125.27, 73.62, 62.45, 21.22. HRMS (Dual ESI): Calcd for $C_{18}H_{18}N_2O_2$ $[M+H]^+$ 295.1368. Found 295.1441.

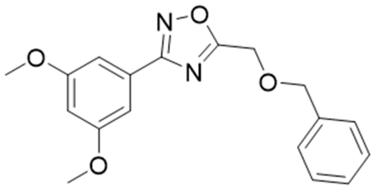

5-((benzyloxy)methyl)-3-(3,5-dimethoxyphenyl)-1,2,4-oxadiazole (**5t**). Yield: 88%. White solid. m.p. 134.4−135.8 °C.1H NMR (500 MHz, CDCl3) δ 7.48 – 7.14 (m, 7H), 6.58 (q, J = 2.2 Hz, 1H), 4.76 (s, 2H), 4.70 (s, 2H), 3.81 (d, J = 2.2 Hz, 6H).13C NMR (500 MHz, CDCl3) δ 175.78, 168.14, 161.26, 136.59, 136.46, 129.23, 128.72, 128.67, 128.34, 128.21, 127.55, 119.25, 115.21, 73.59, 70.11, 62.45. HRMS (Dual ESI): Calcd for $C_{18}H_{19}N_2O_4$ $[M+H]^+$ 327.1267. Found 327.1344.

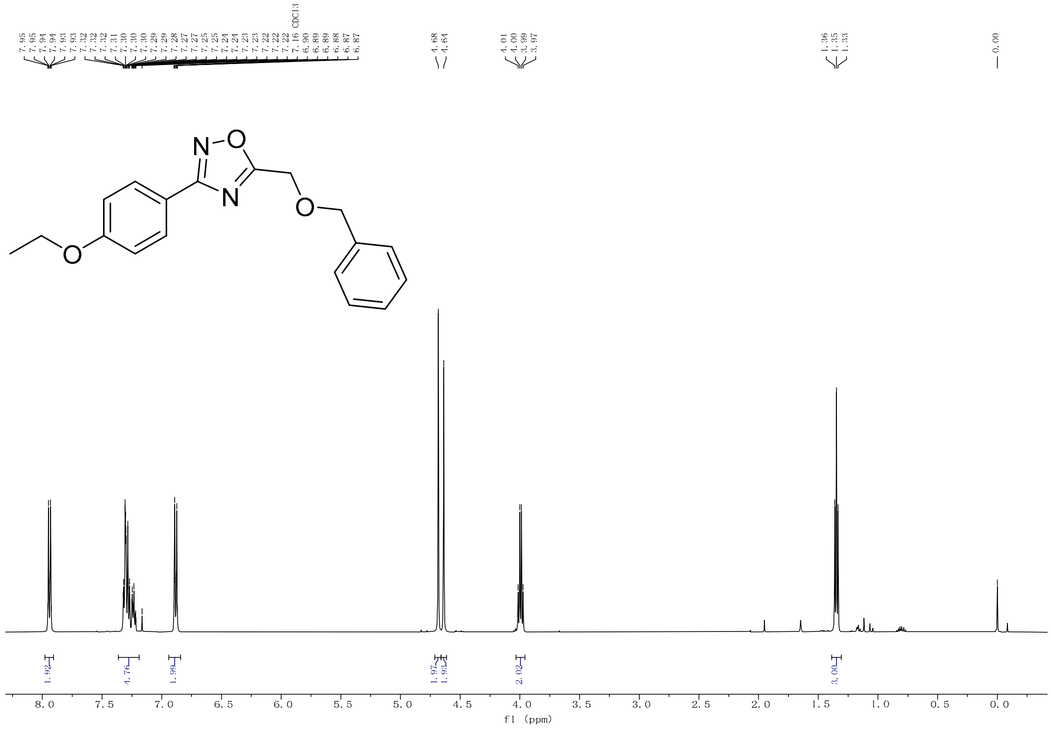

Figure S1A. ^{1}H NMR for compound **5a**

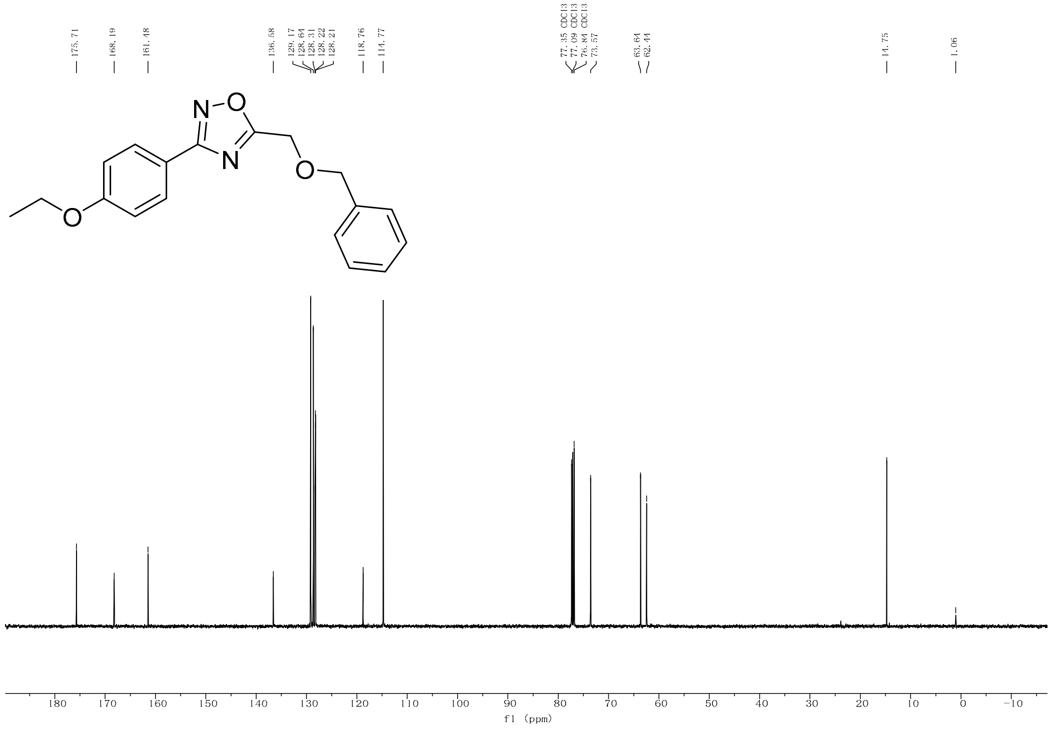

Figure S1B. ^{13}C NMR for compound **5a**

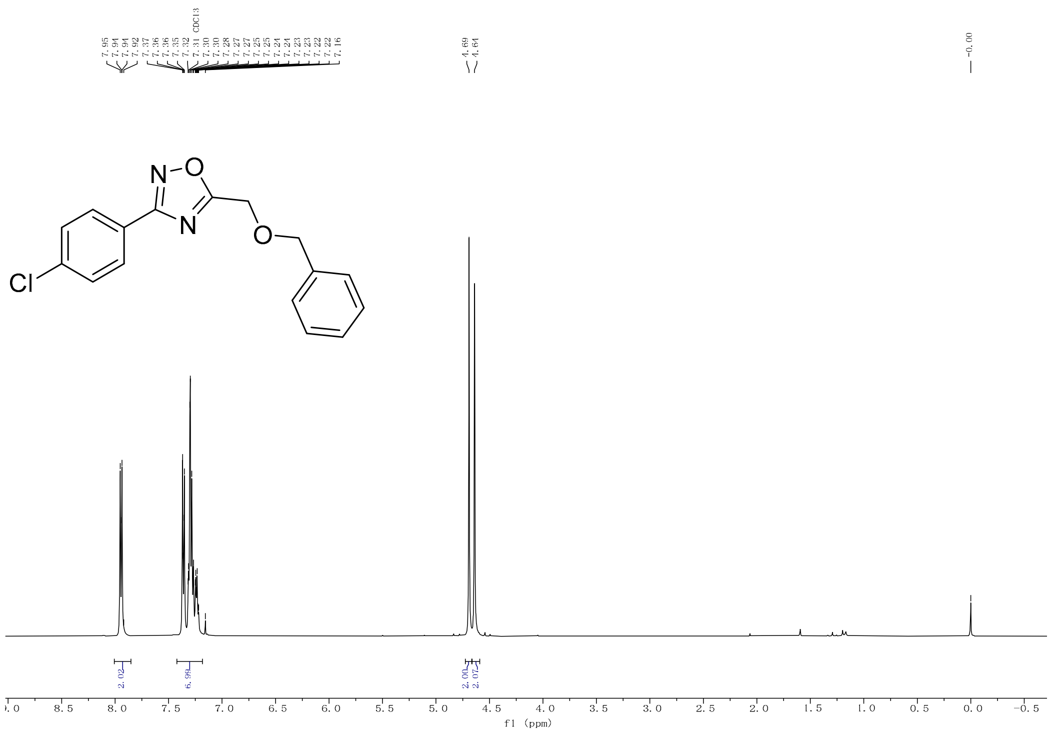

Figure S2A. ^{1}H NMR for compound **5b**

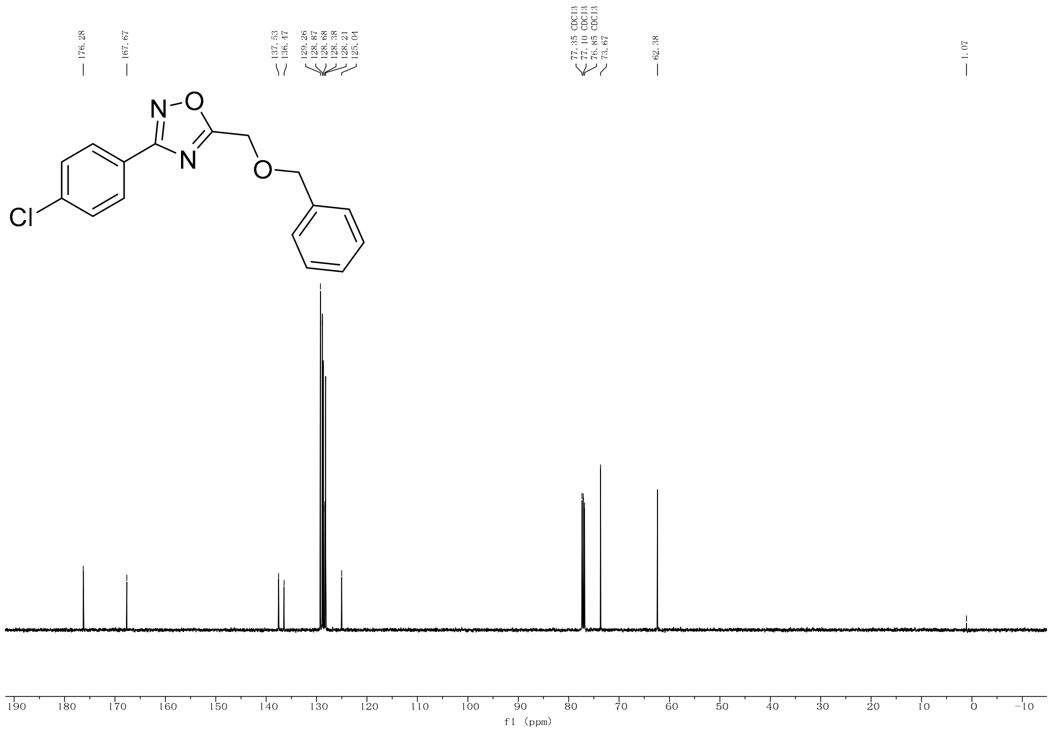

Figure S2B. ^{13}C NMR for compound **5b**

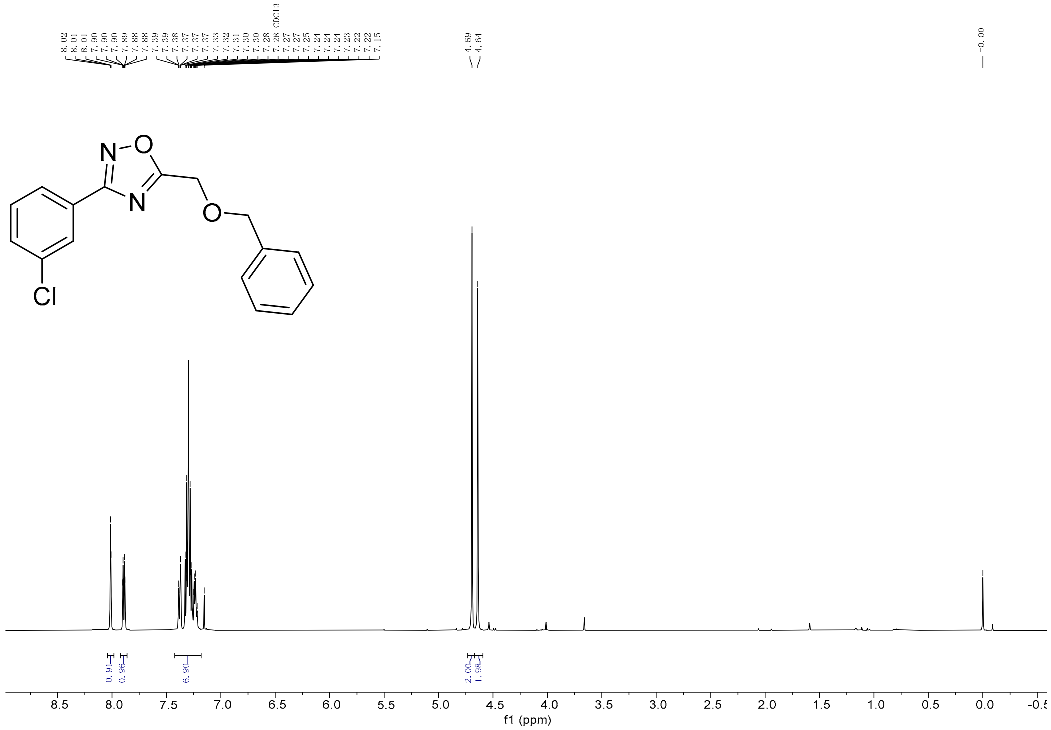

Figure S3A. ^{1}H NMR for compound **5c**

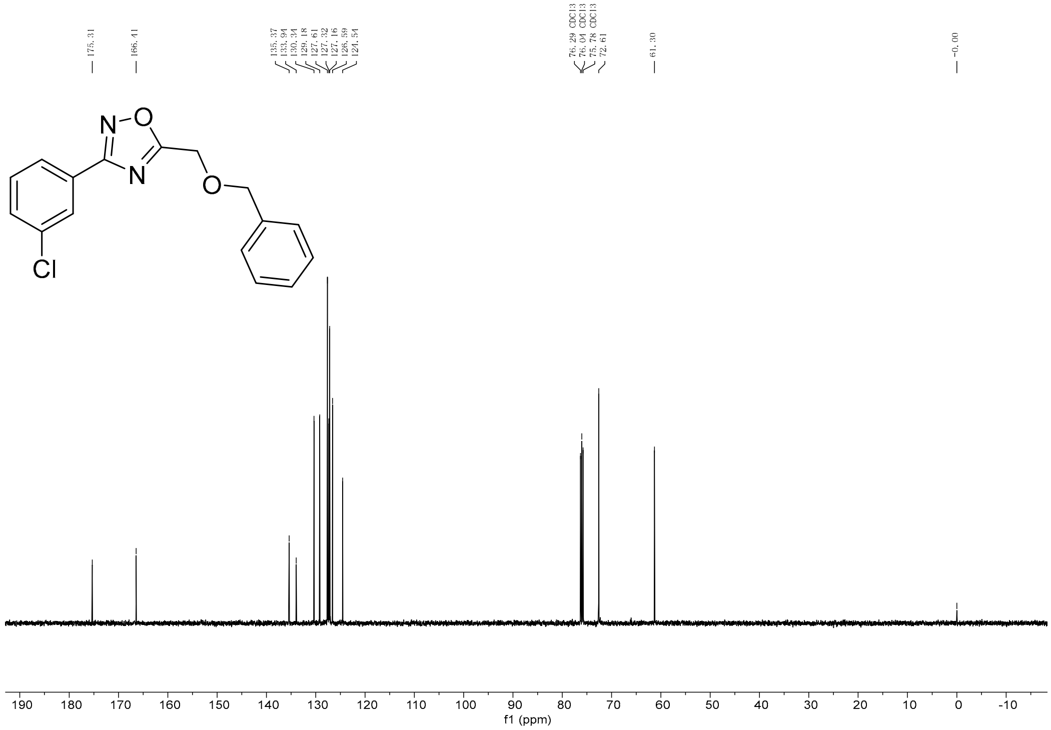

Figure S3B. ^{13}C NMR for compound **5c**

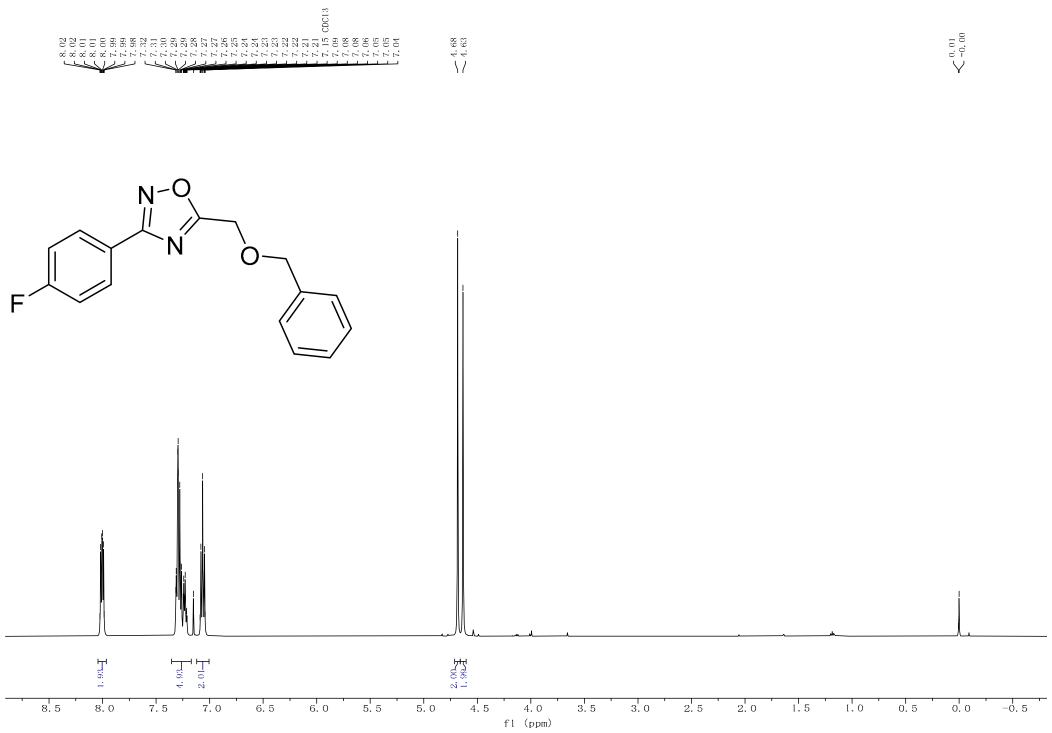

Figure S4A. ^{1}H NMR for compound **5d**

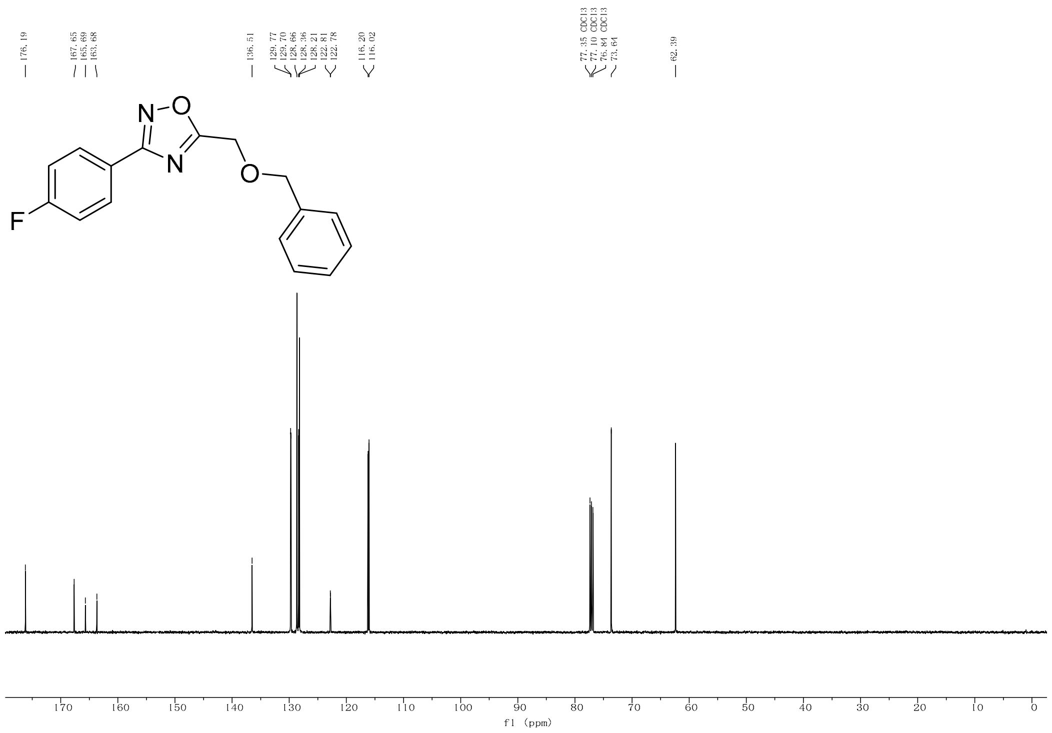

Figure S4B. ^{13}C NMR for compound **5d**

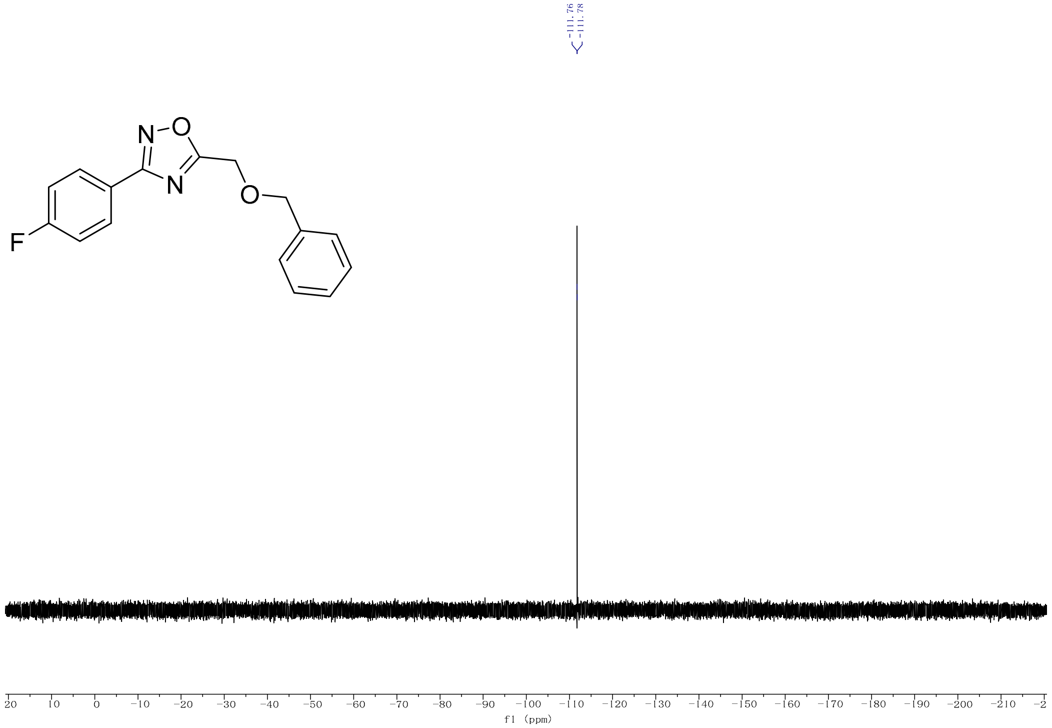

Figure S4C. ^{19}F NMR for compound **5d**

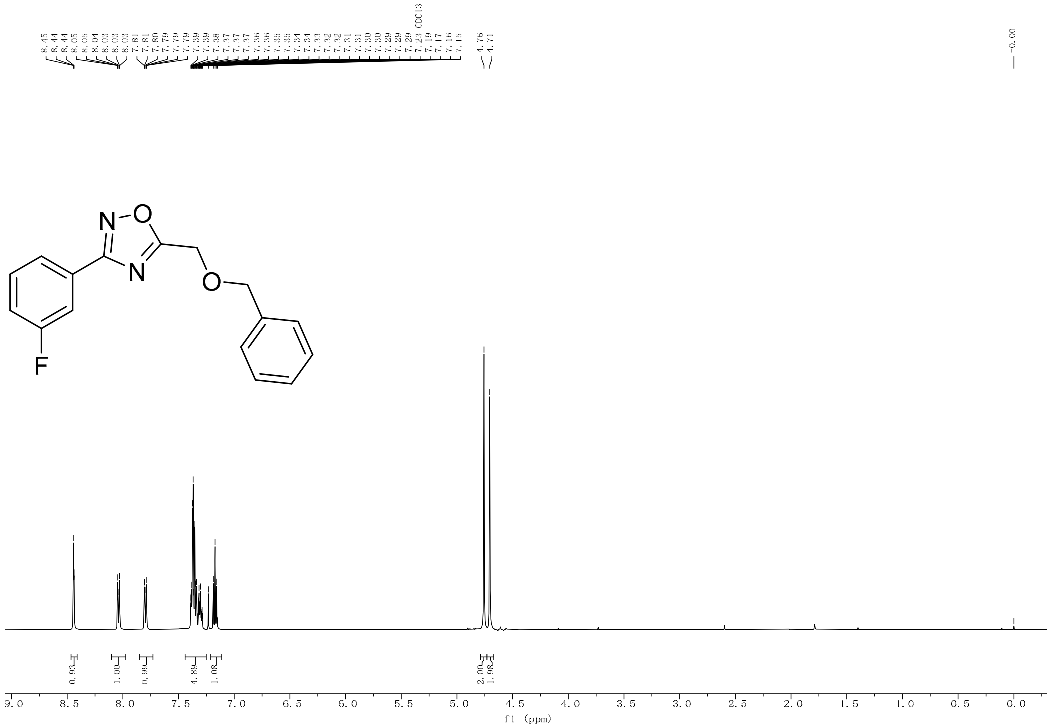

Figure S5A. ^{1}H NMR for compound **5e**

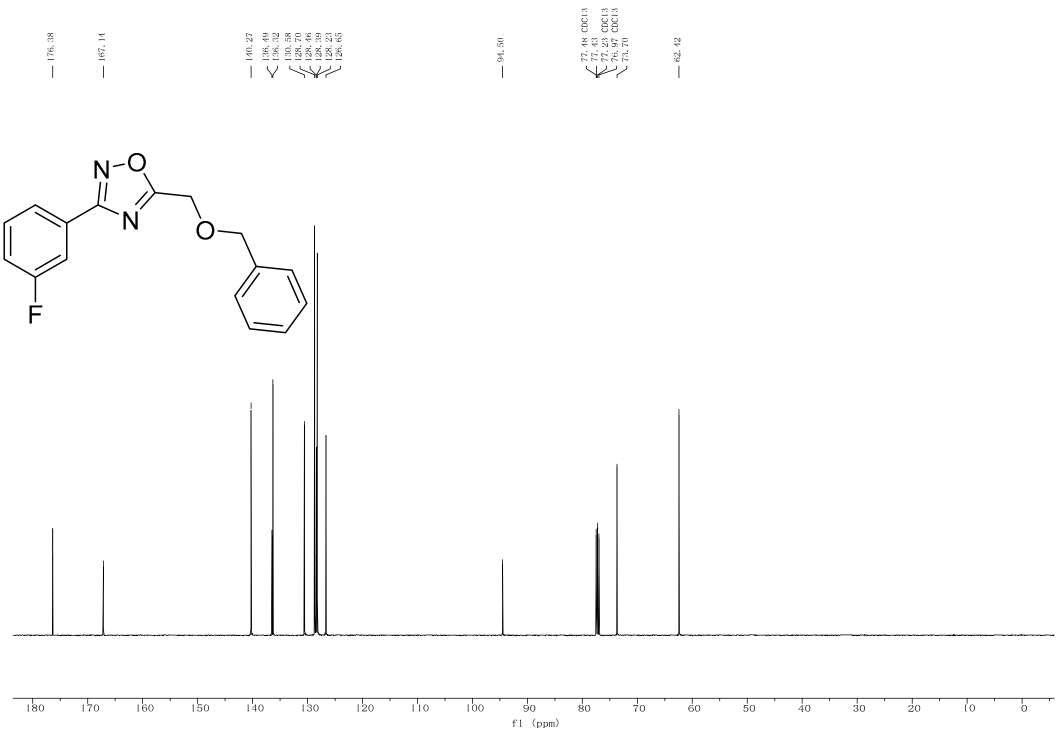

Figure S5B. ^{13}C NMR for compound **5e**

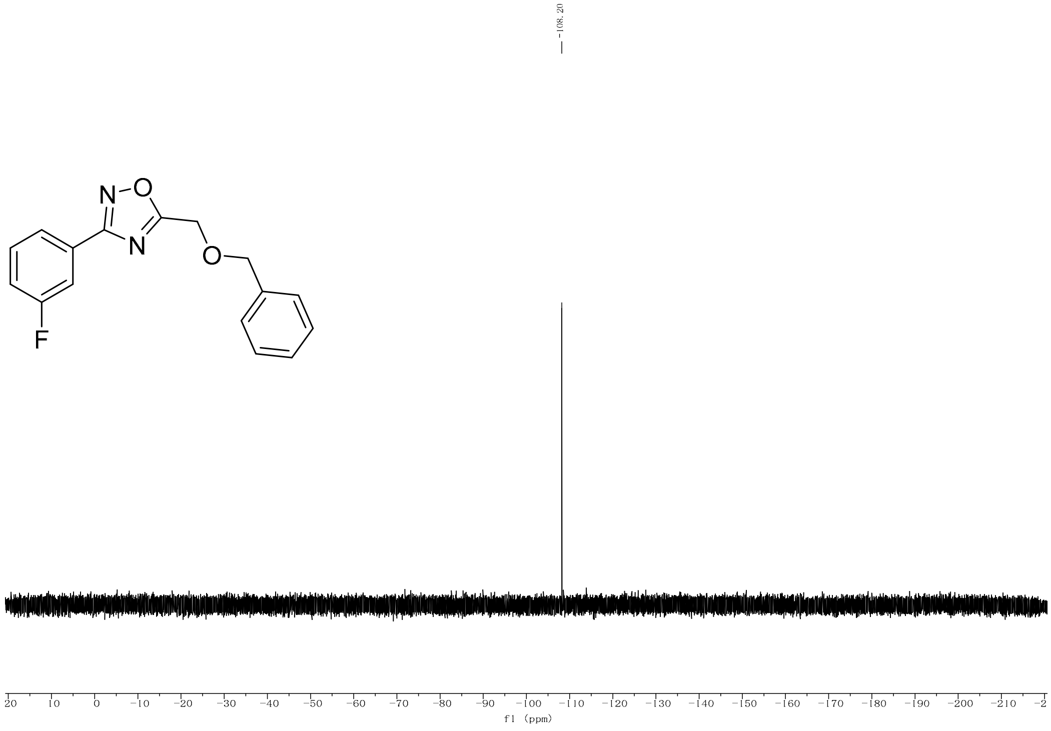

Figure S5C. ^{19}F NMR for compound **5e**

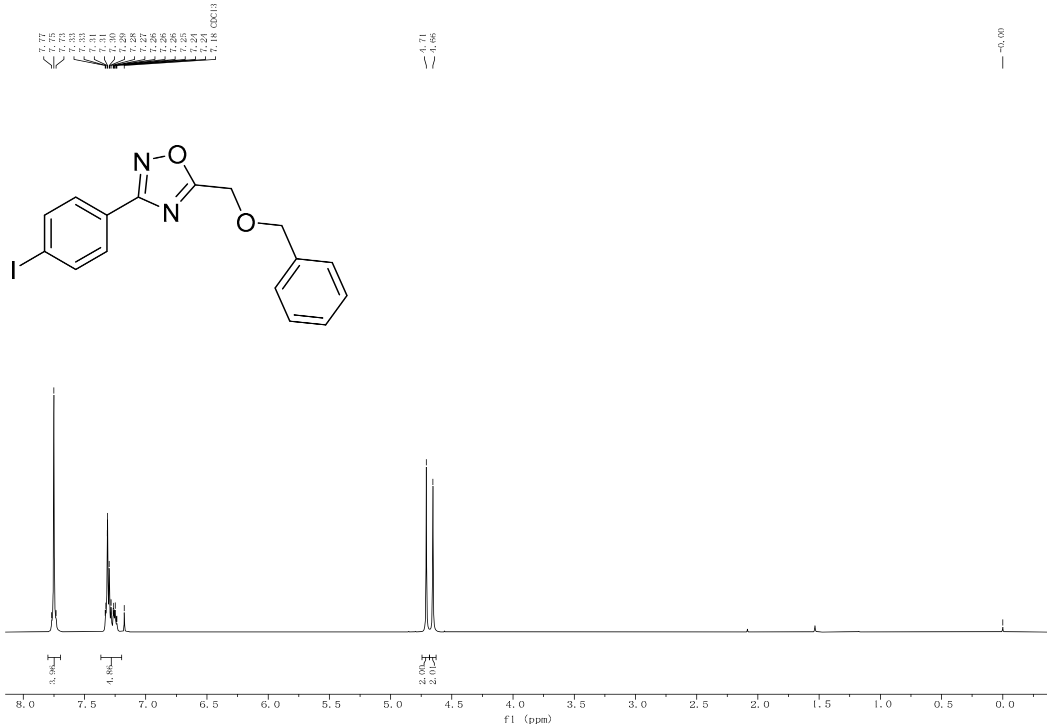

Figure S6A. ^{1}H NMR for compound **5f**

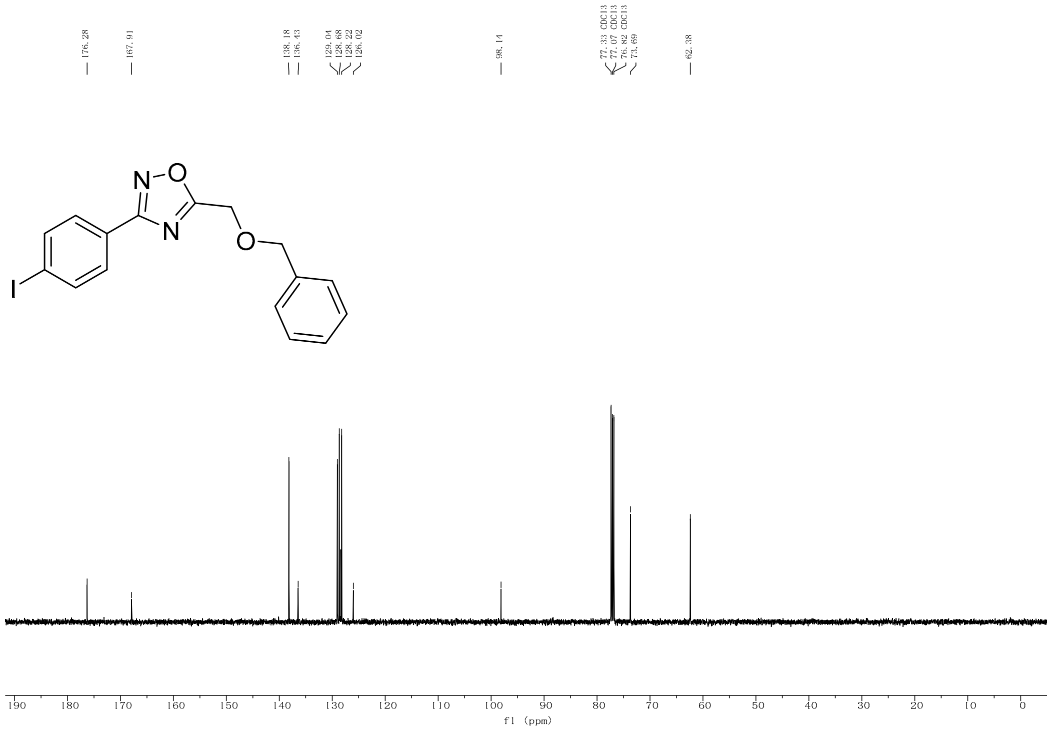

Figure S6B. ^{13}C NMR for compound **5f**

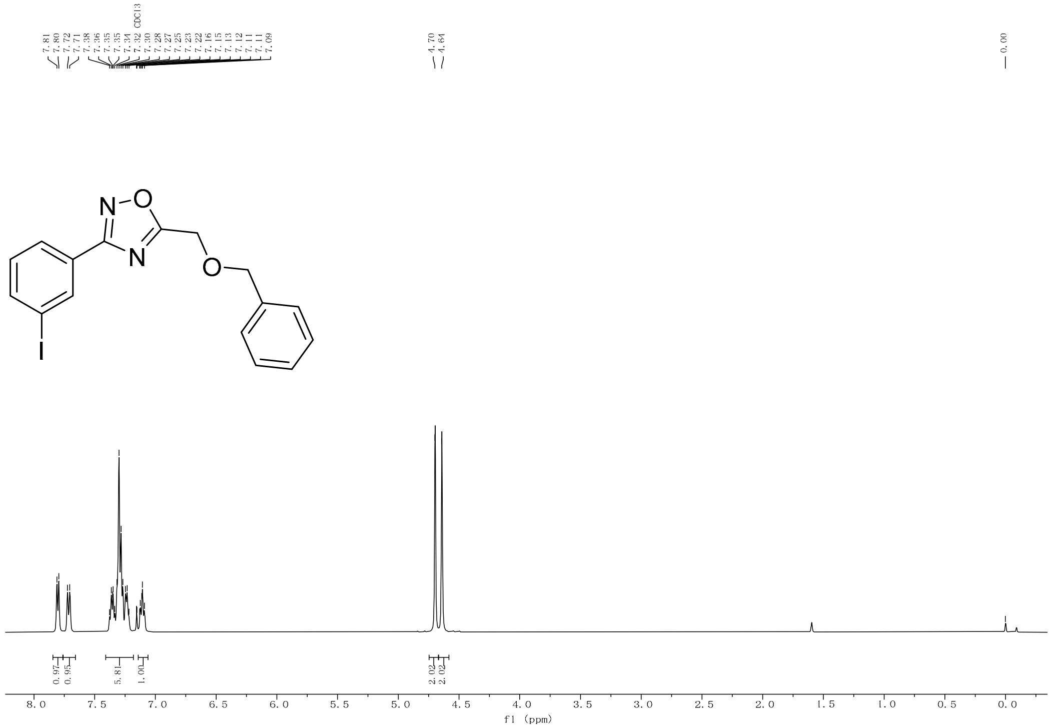

Figure S7A. ^{1}H NMR for compound **5g**

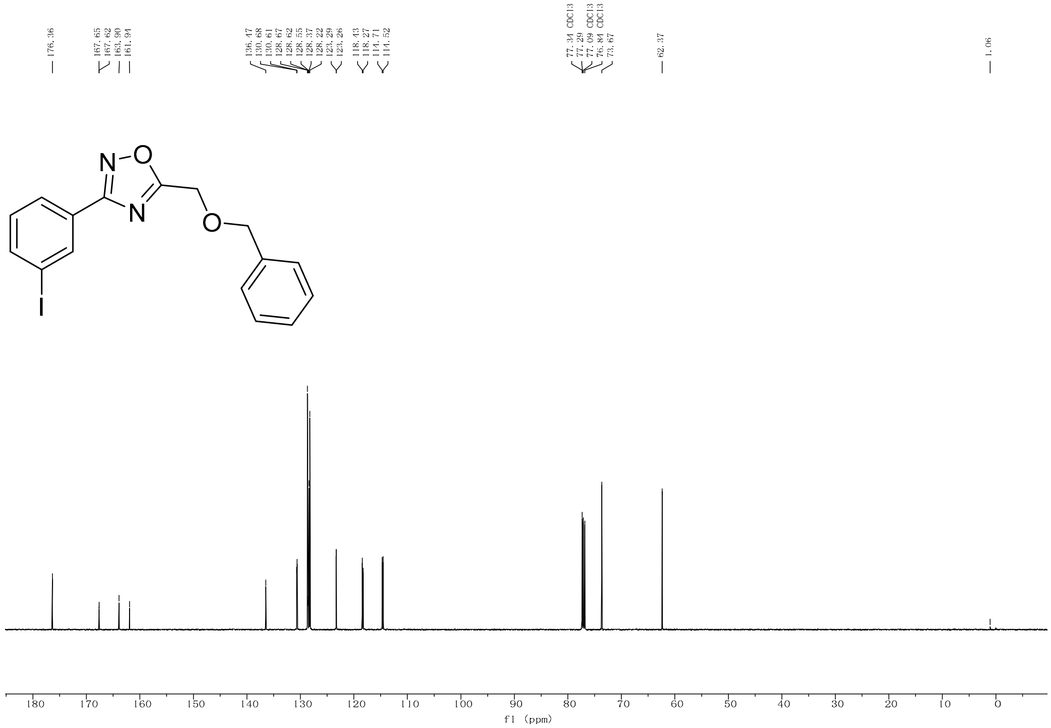

Figure S7B. ^{13}C NMR for compound **5g**

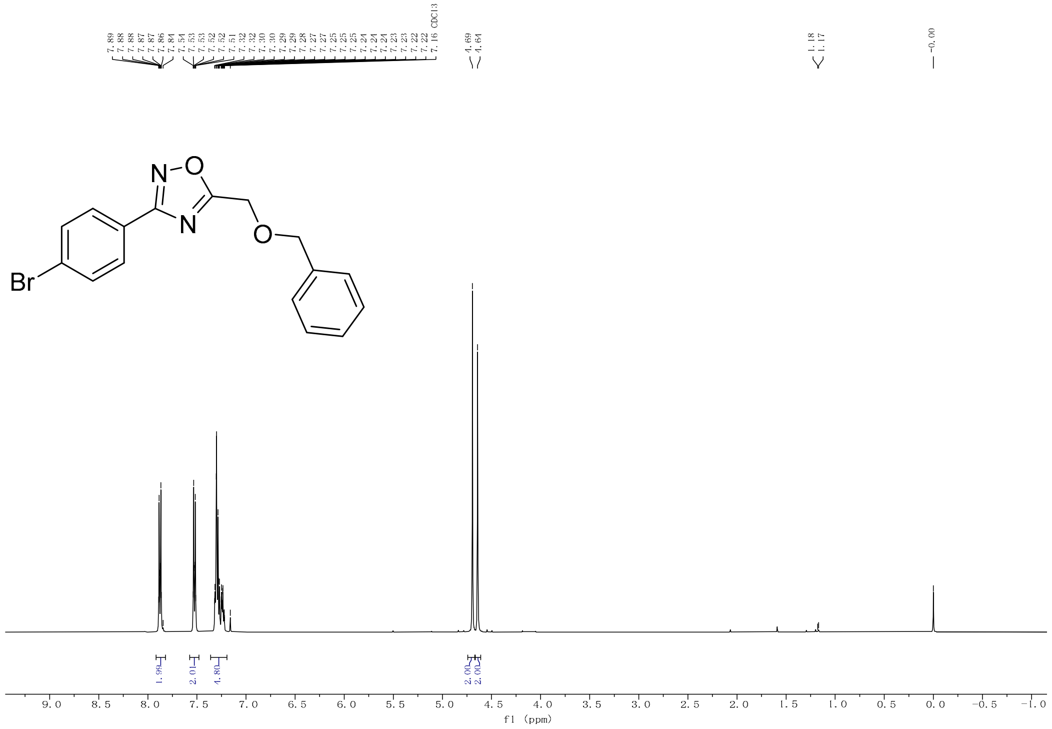

Figure S8A. ^{1}H NMR for compound **5h**

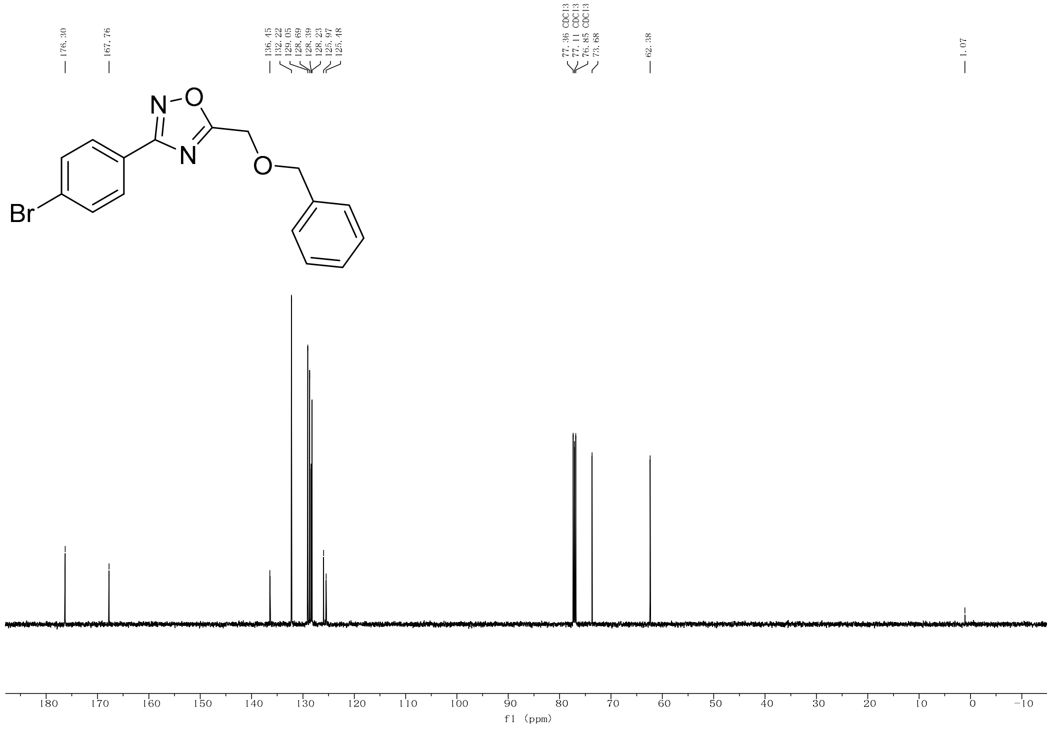

Figure S8B. ^{13}C NMR for compound **5h**

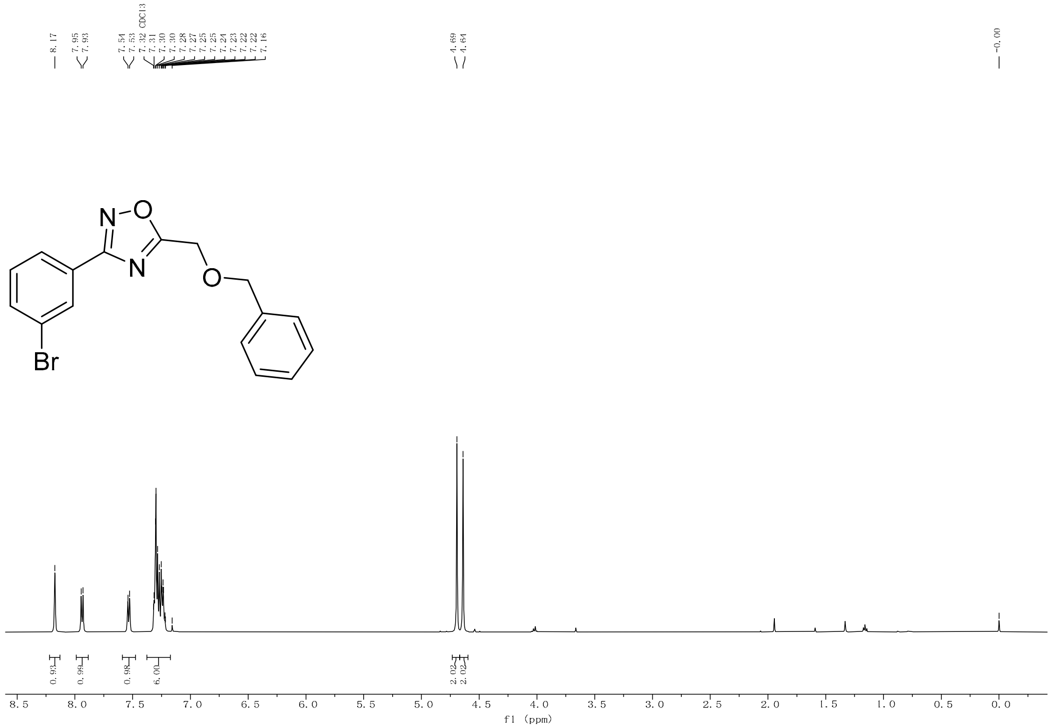

Figure S9A. ^{1}H NMR for compound **5i**

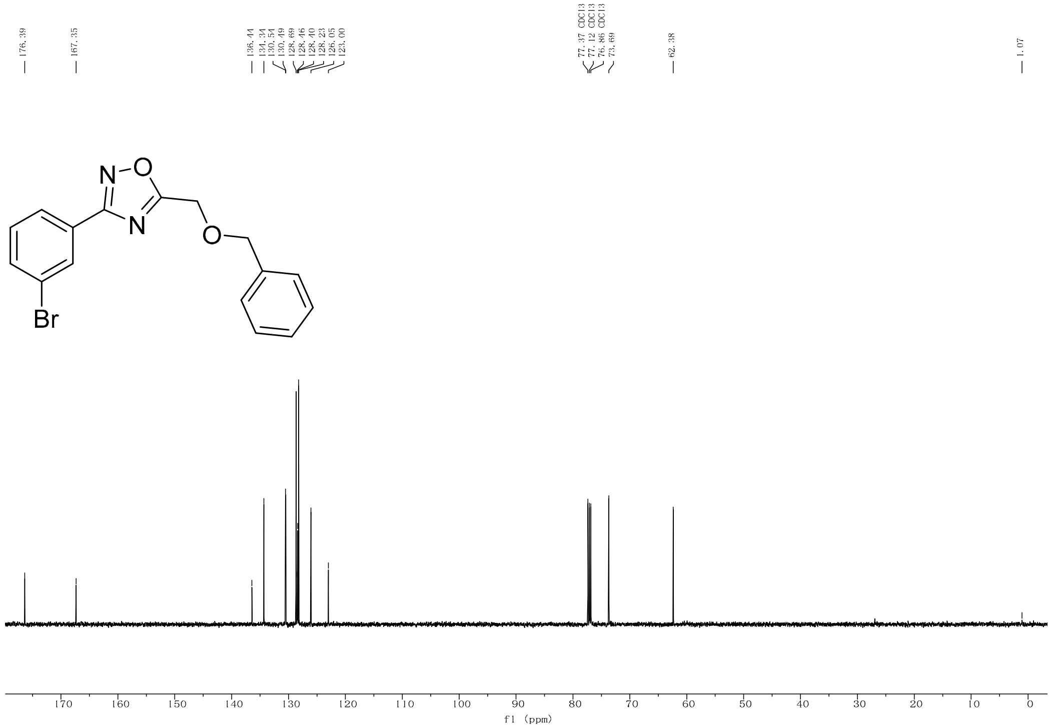

Figure S9B. ^{13}C NMR for compound **5i**

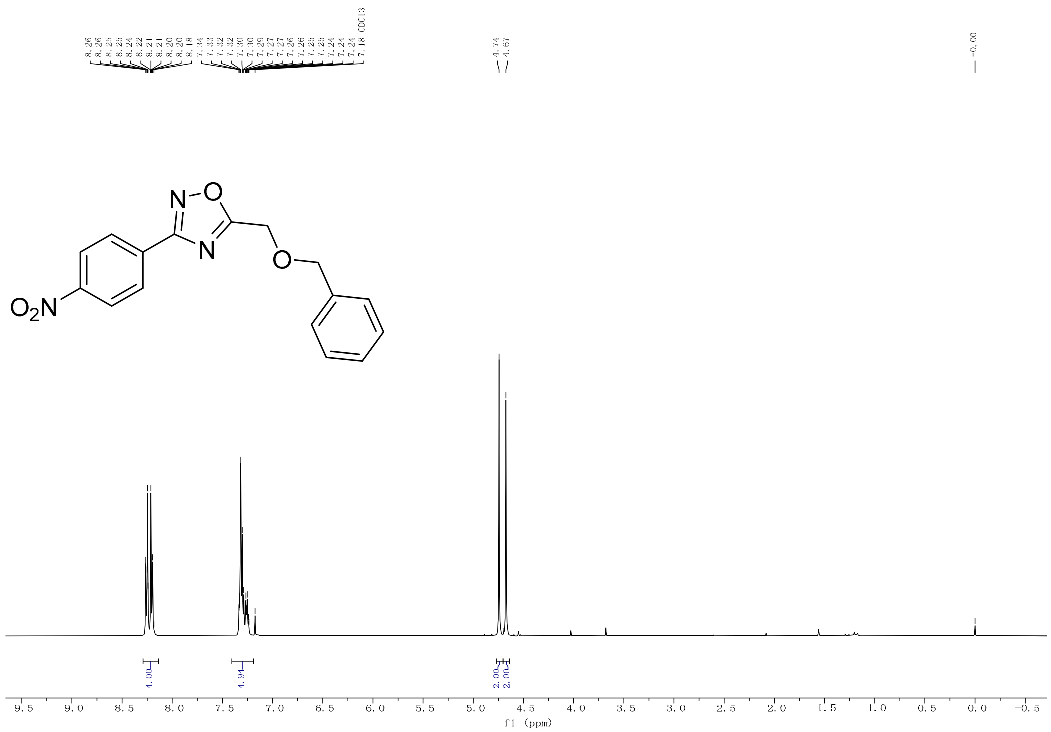

Figure S10A. ^{1}H NMR for compound **5j**

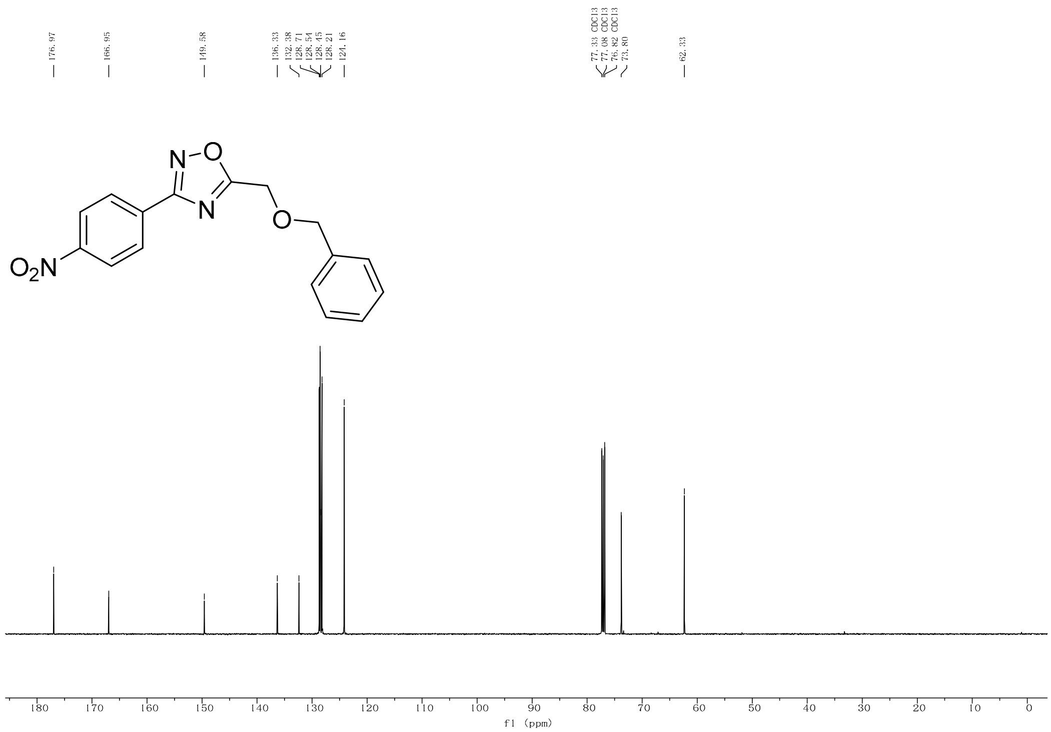

Figure S10B. ^{13}C NMR for compound **5j**

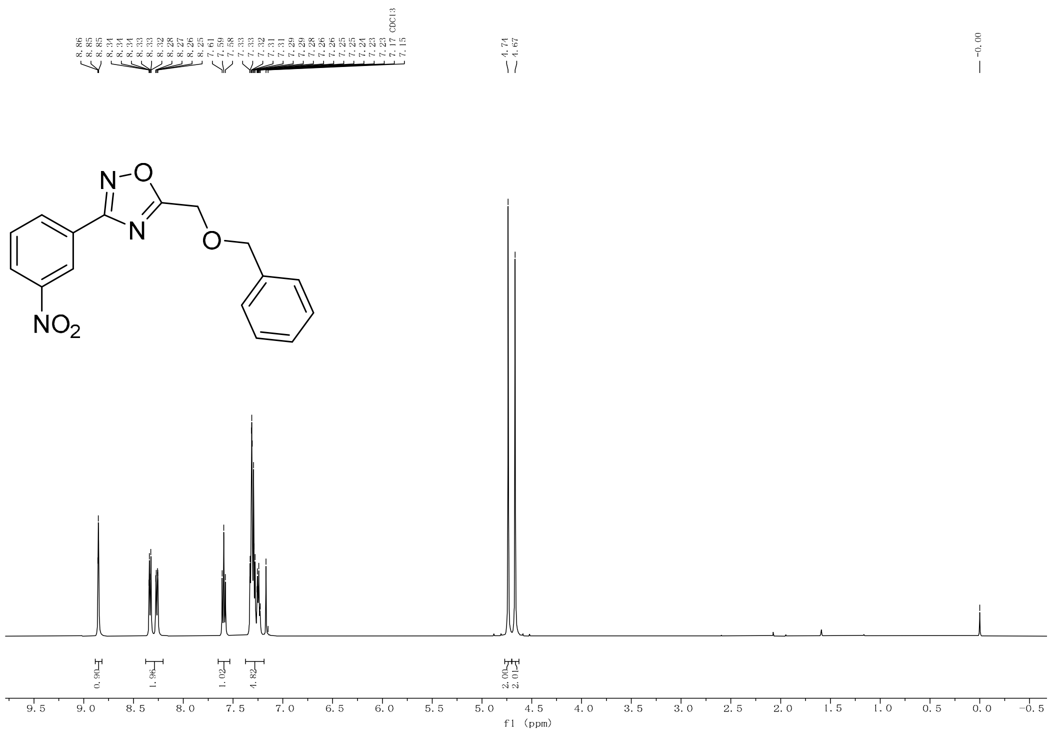

Figure S11A. ^{1}H NMR for compound **5k**

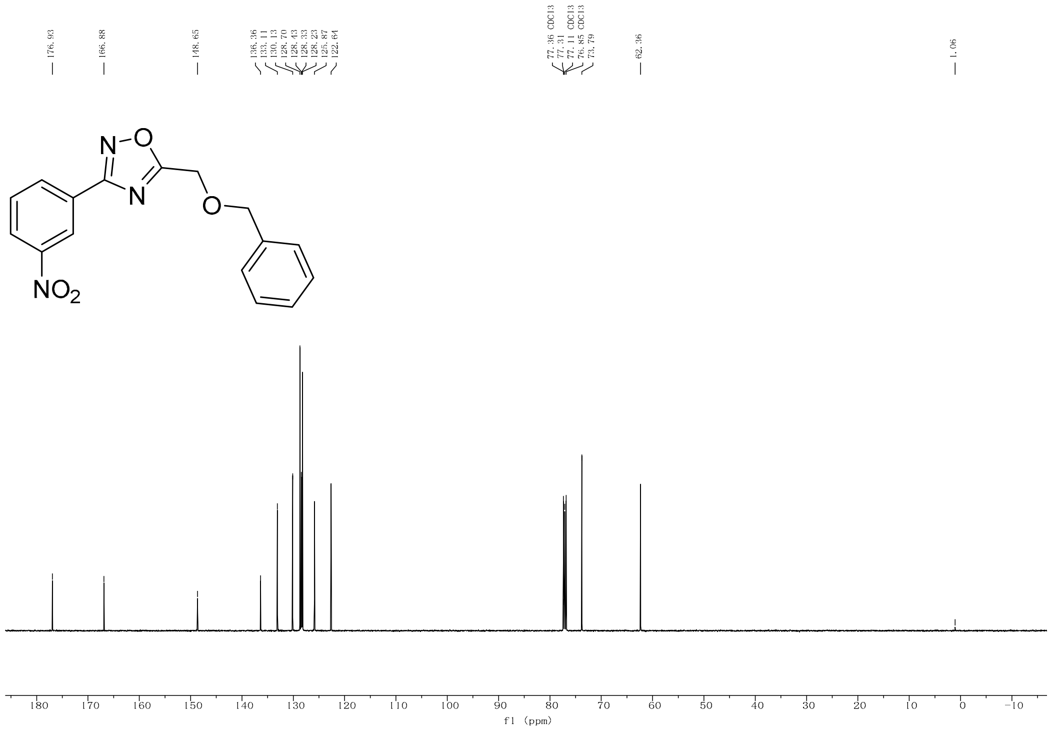

Figure S11B. ^{13}C NMR for compound **5k**

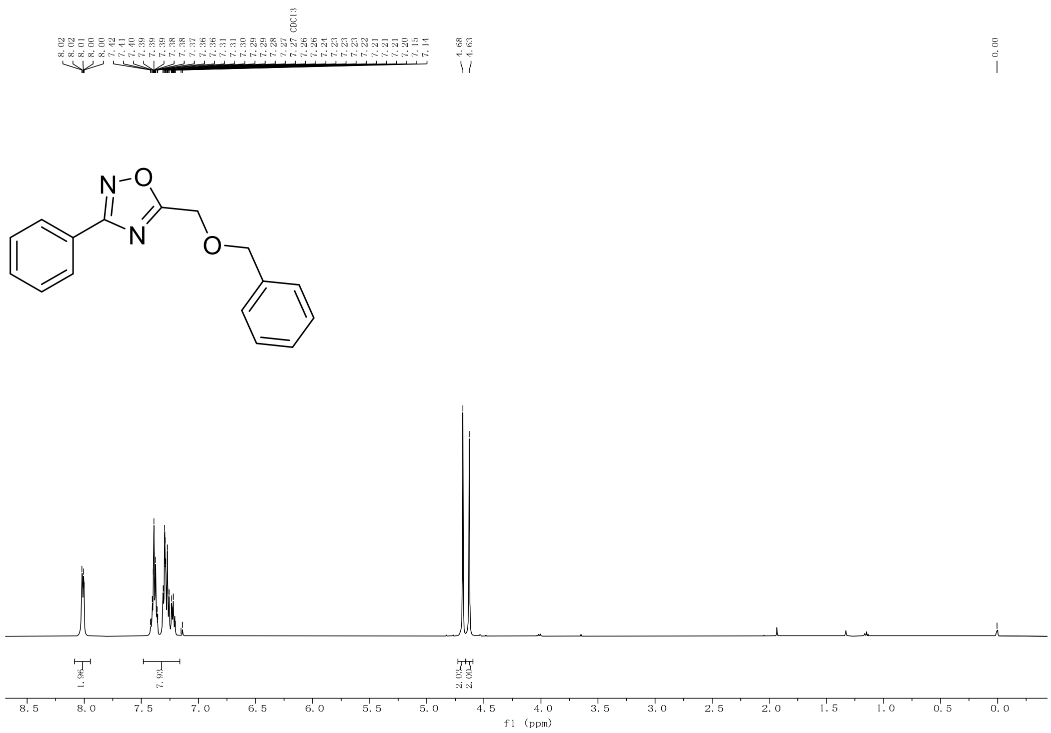

Figure S12A. ^{1}H NMR for compound **5l**

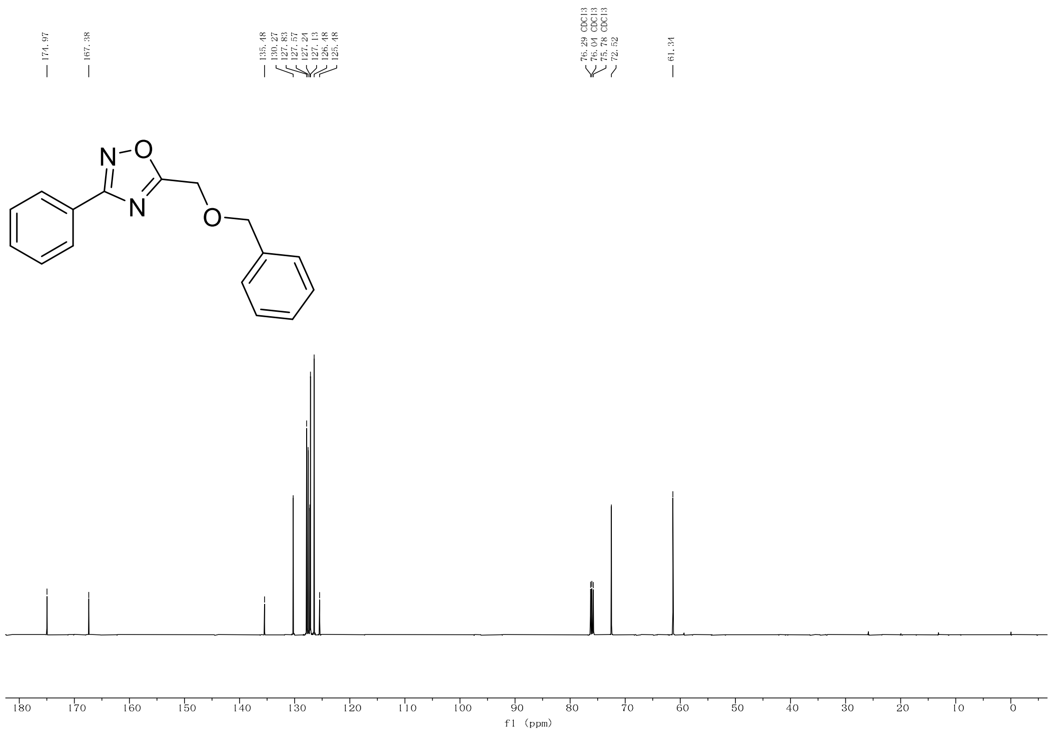

Figure S12B. ^{13}C NMR for compound **5l**

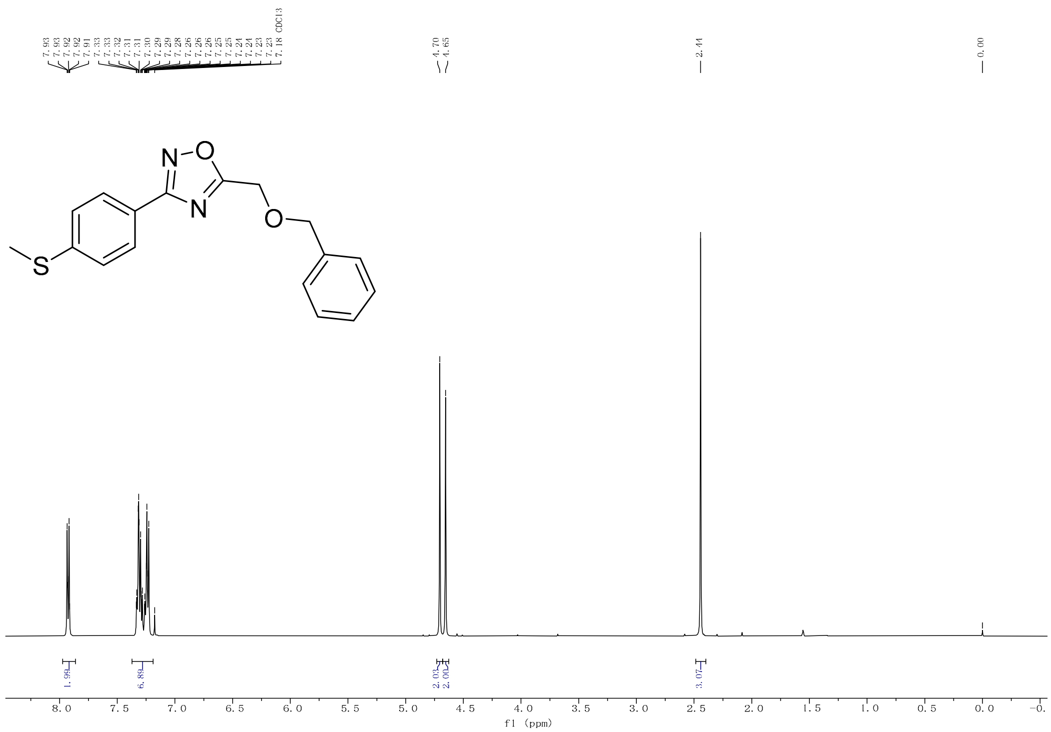

Figure S13A. ^{1}H NMR for compound **5m**

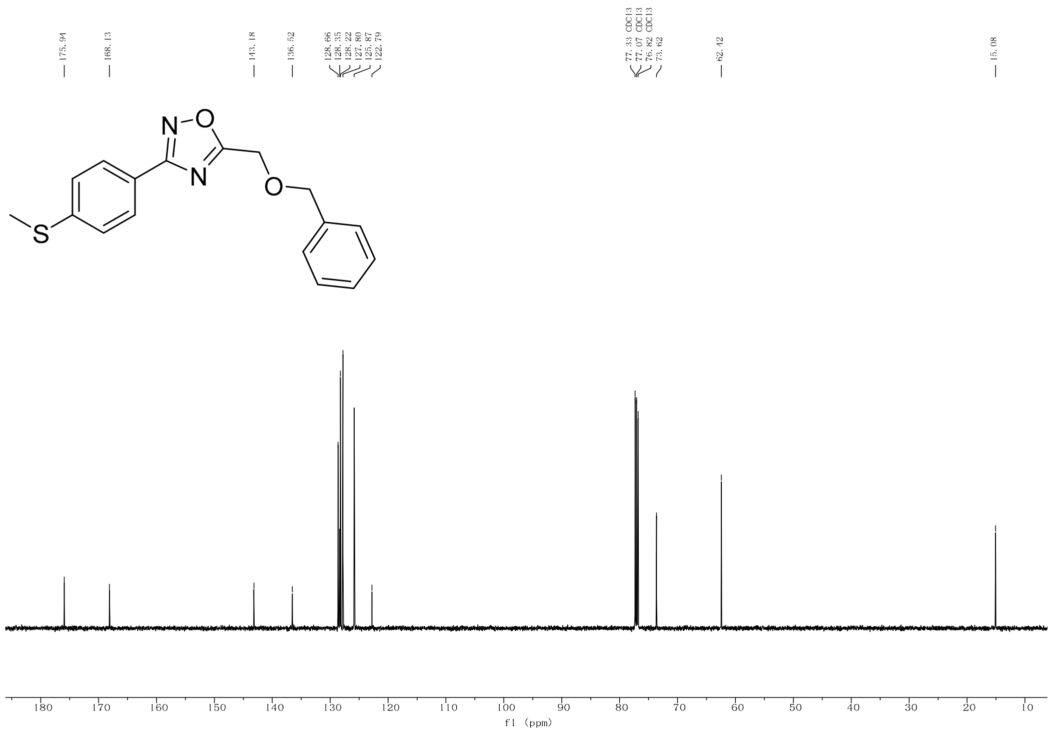

Figure S13B. ^{13}C NMR for compound **5m**

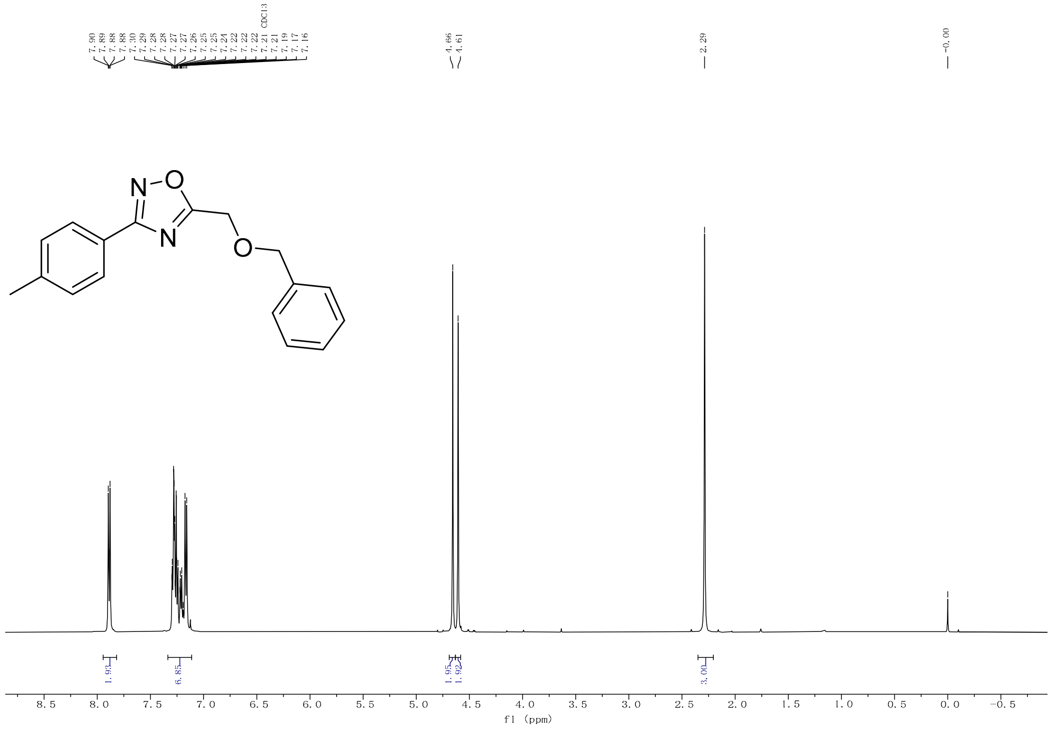

Figure S14A. ^{1}H NMR for compound **5n**

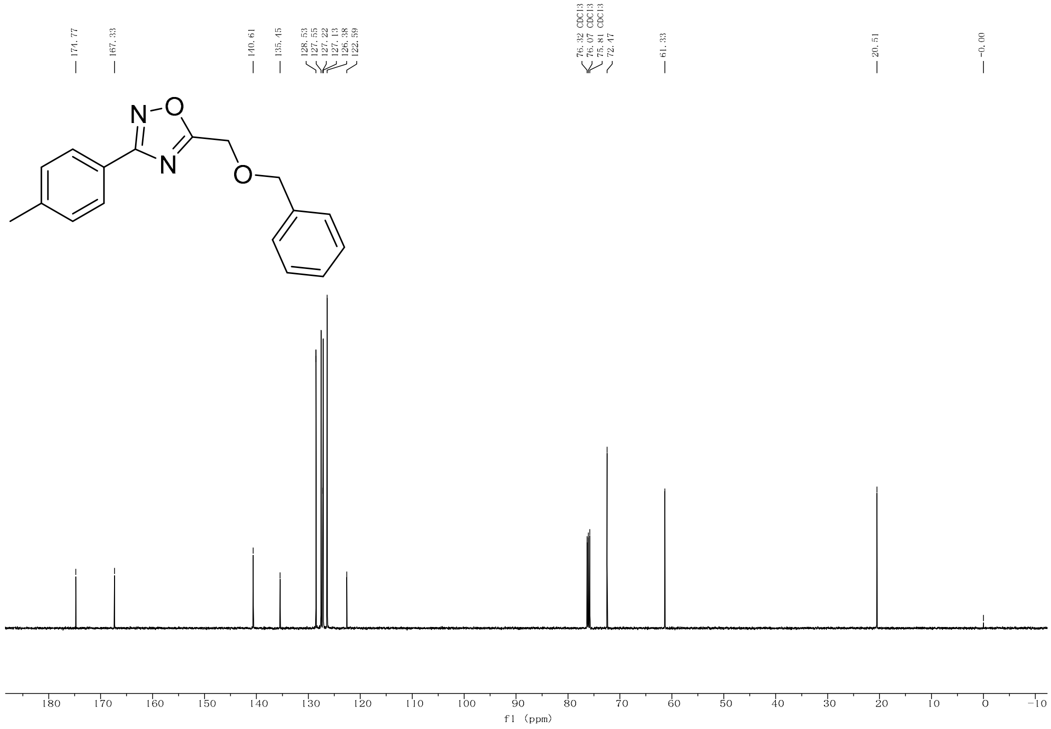

Figure S14B. ^{13}C NMR for compound **5n**

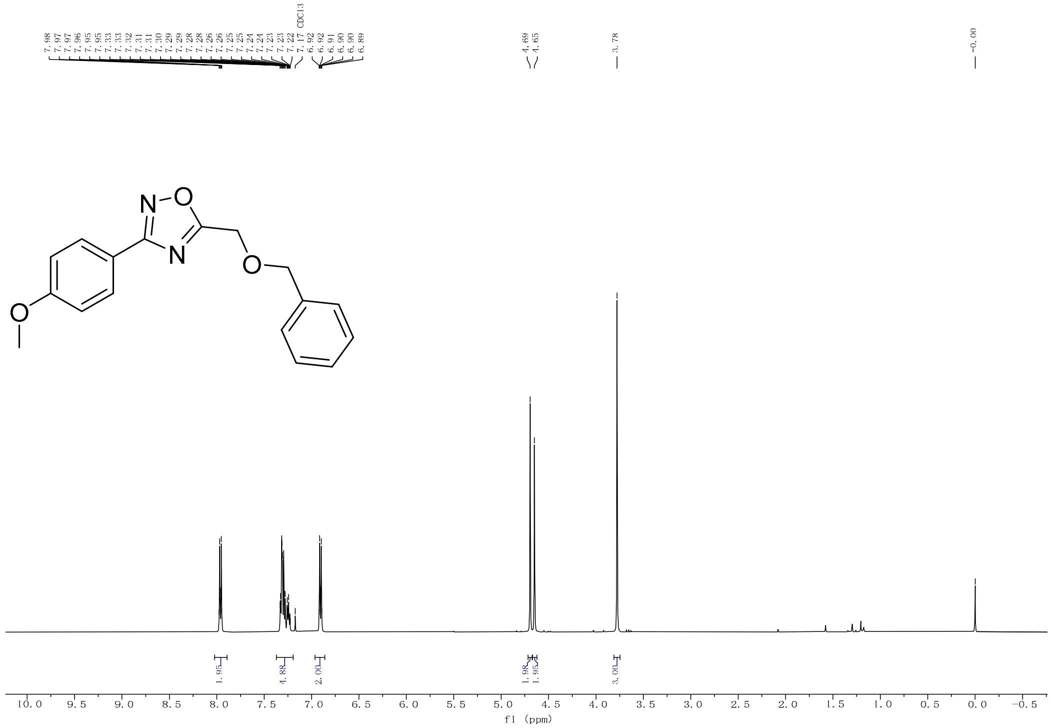

Figure S15A. ^{1}H NMR for compound **5o**

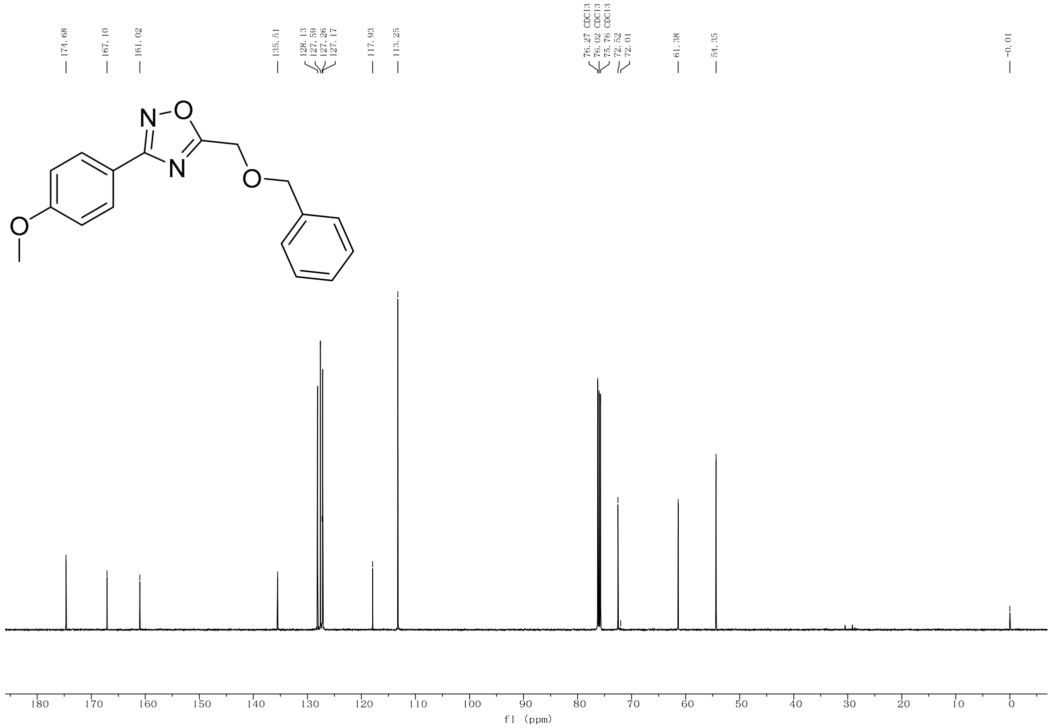

Figure S15B. ^{13}C NMR for compound **5o**

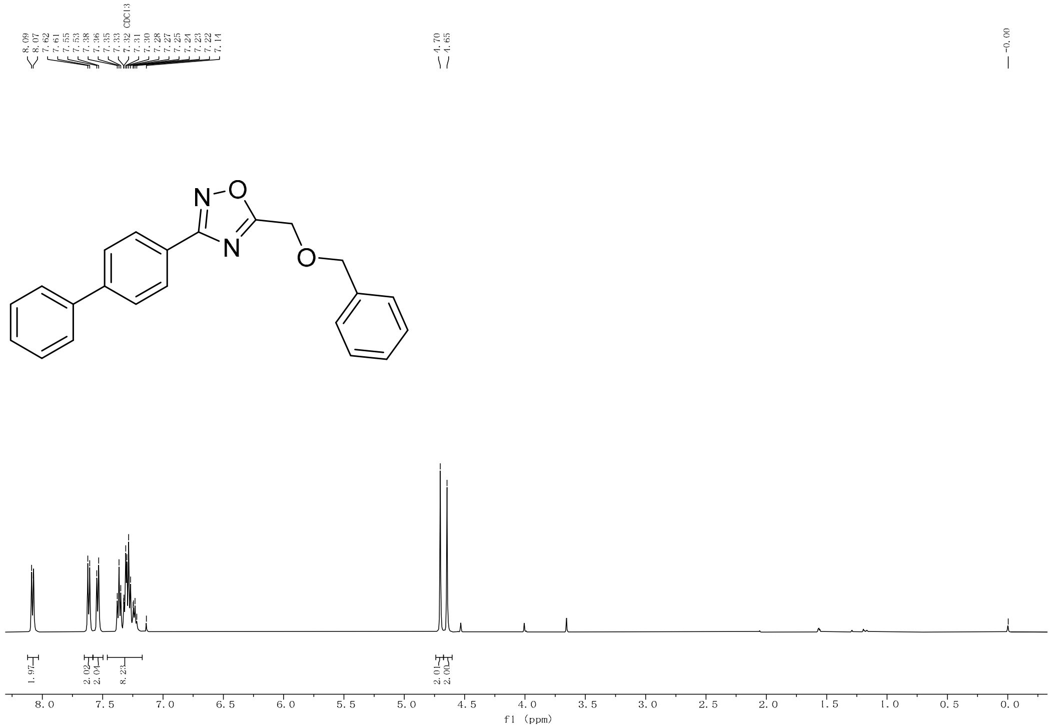

Figure S16A. ^{1}H NMR for compound **5p**

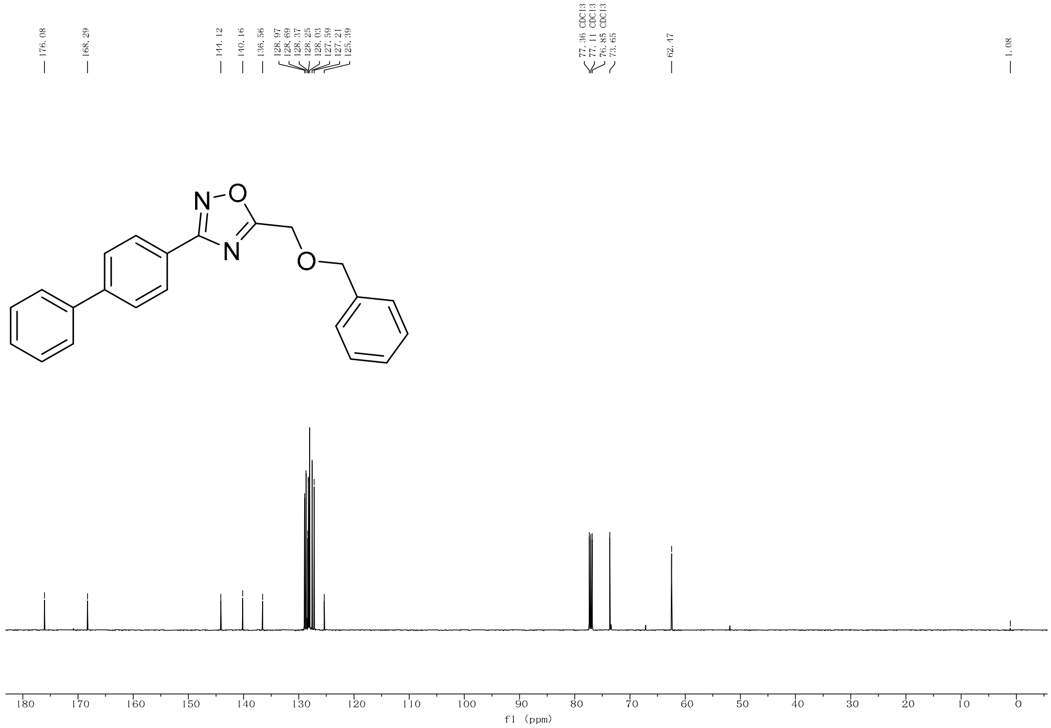

Figure S16B. ^{13}C NMR for compound **5p**

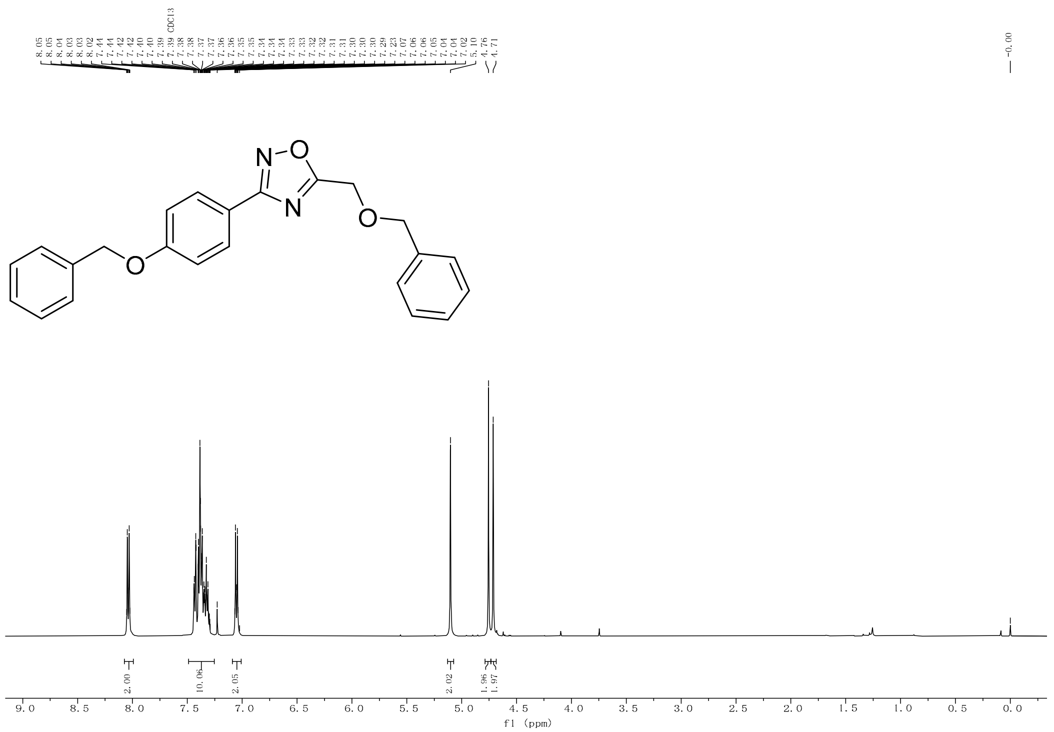

Figure S17A. ^{1}H NMR for compound **5q**

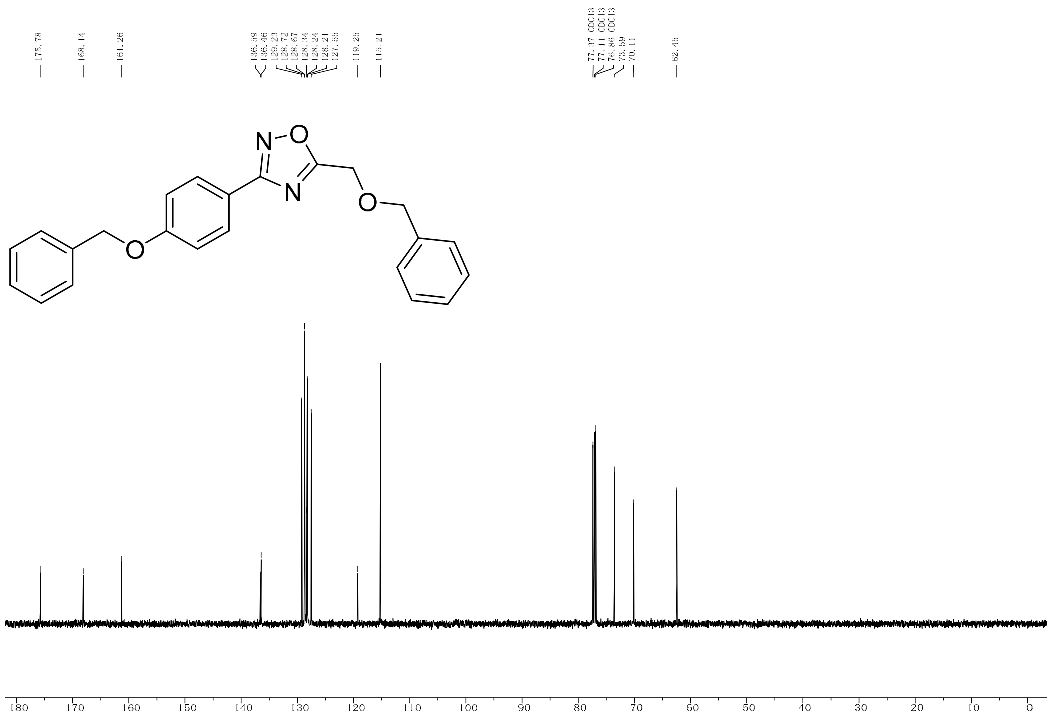

Figure S17B. ^{13}C NMR for compound **5q**

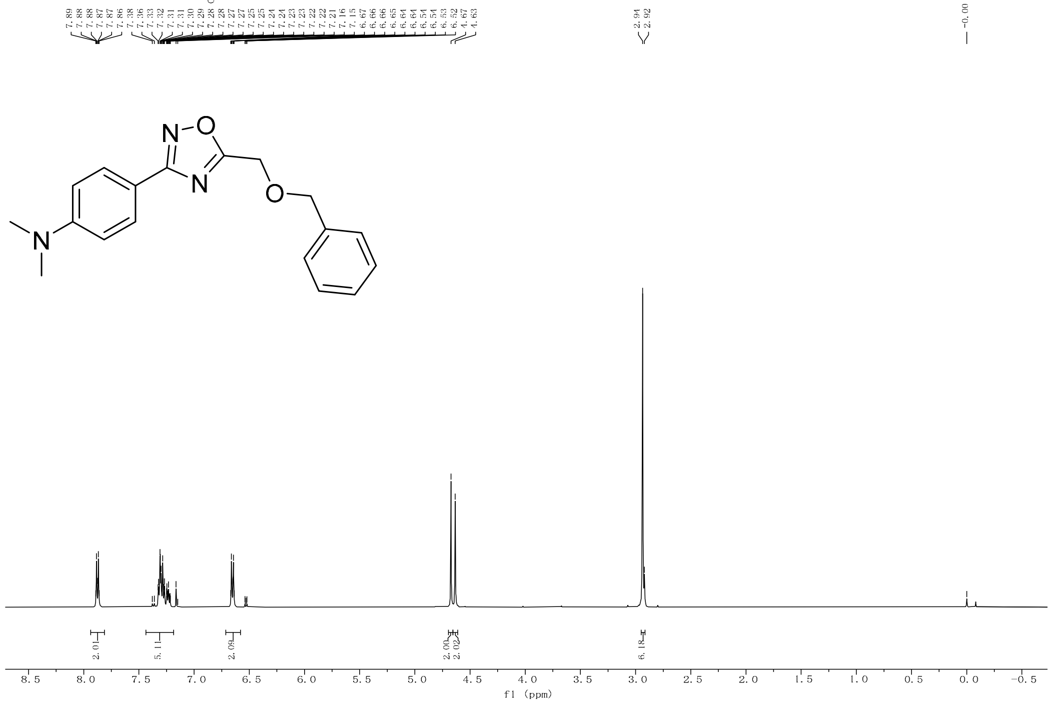

Figure S18A. ^{1}H NMR for compound **5r**

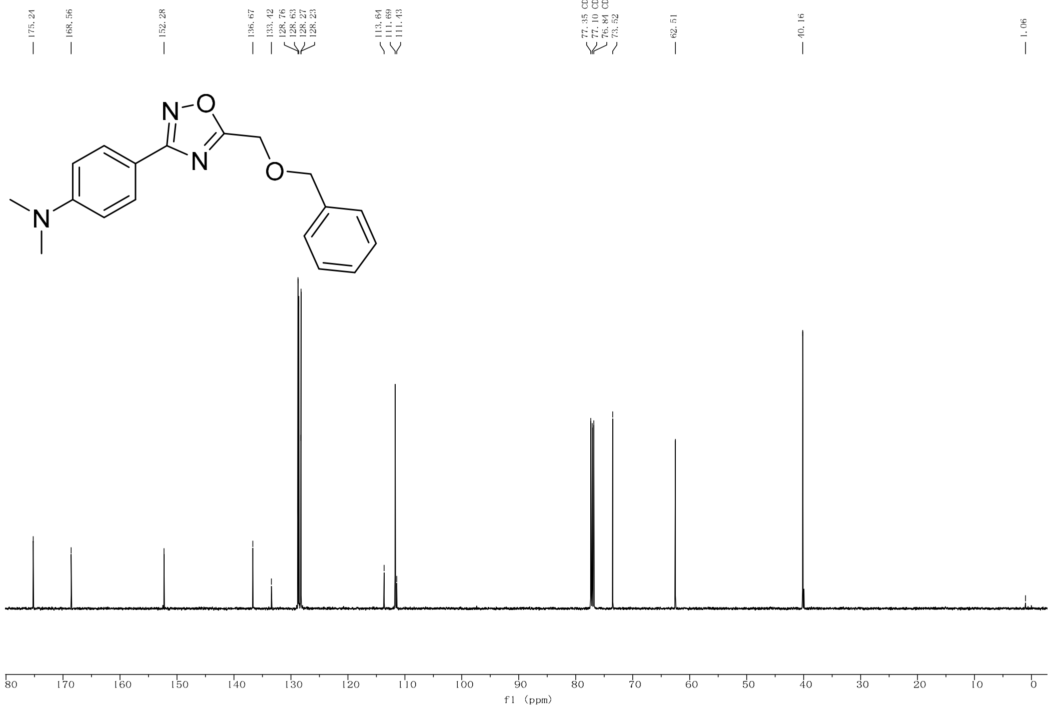

Figure S18B. ^{13}C NMR for compound **5r**

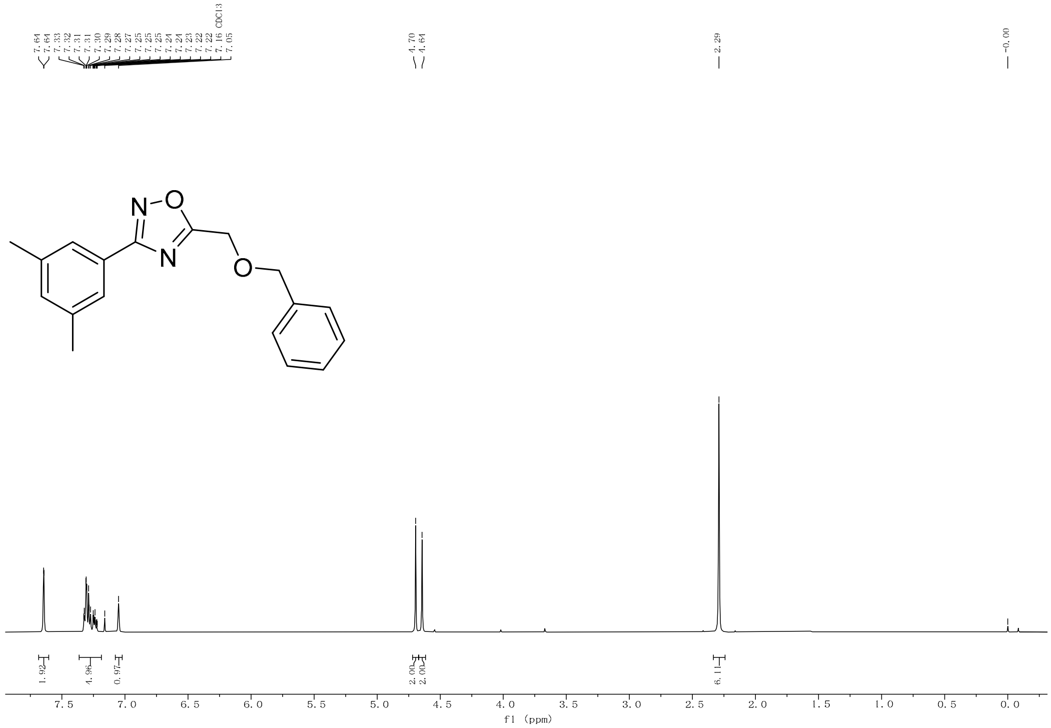

Figure S19A. ^{1}H NMR for compound **5s**

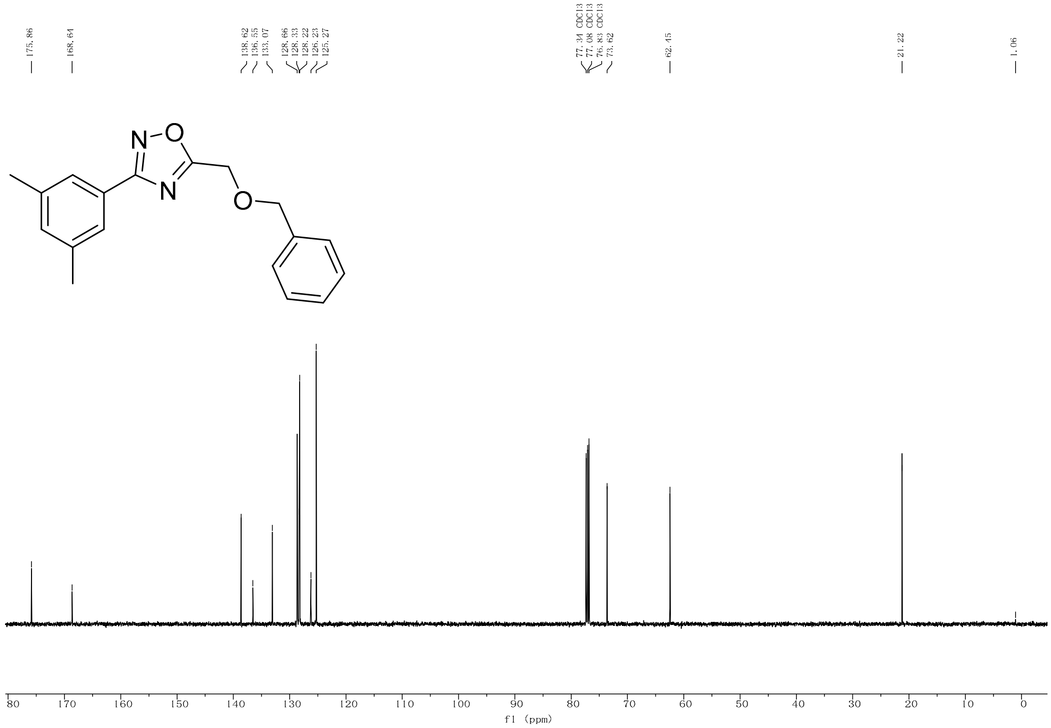

Figure S19B. ^{13}C NMR for compound **5s**

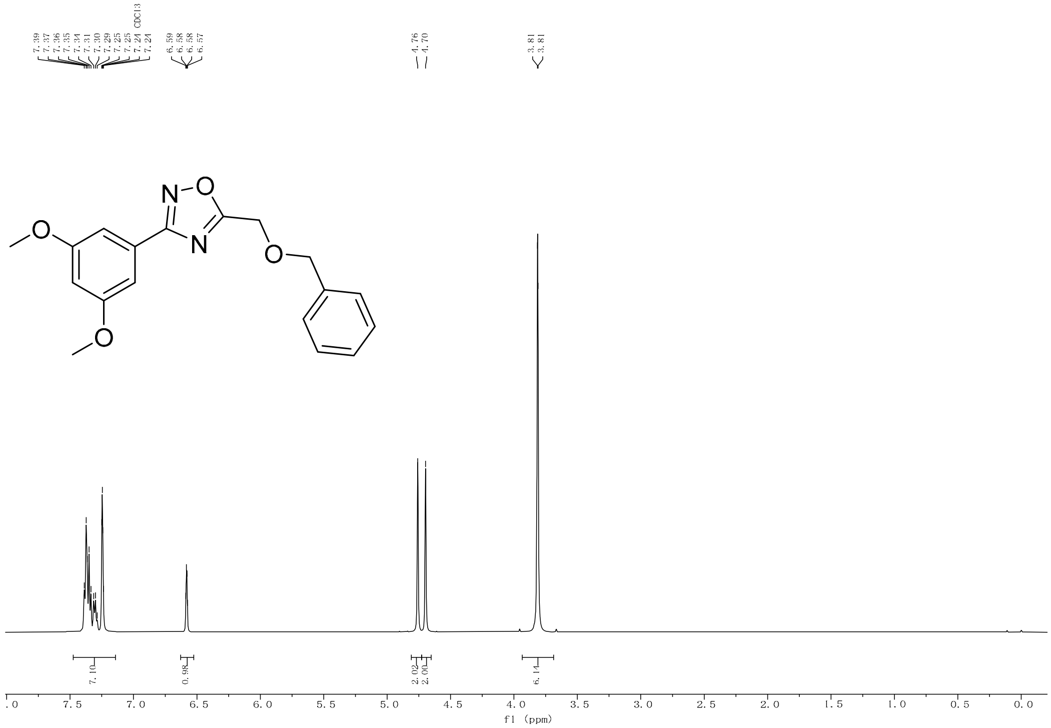

Figure S20A. ^{1}H NMR for compound **5t**

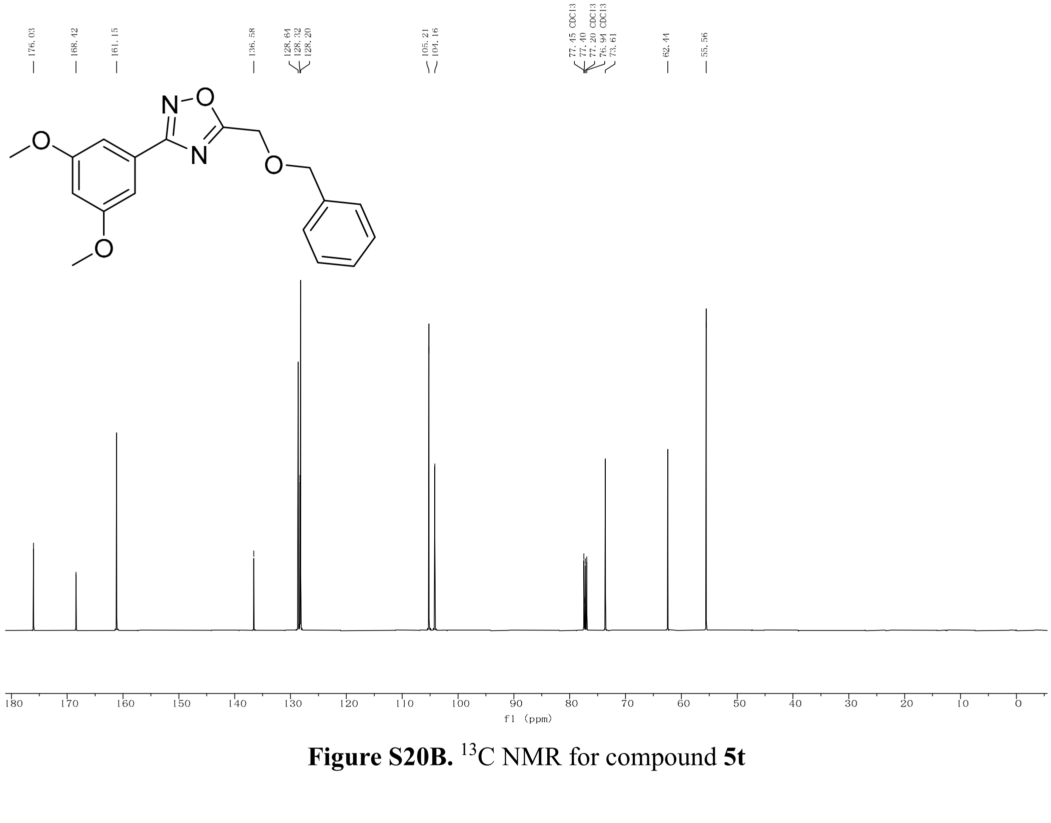

Figure S20B. ^{13}C NMR for compound **5t**

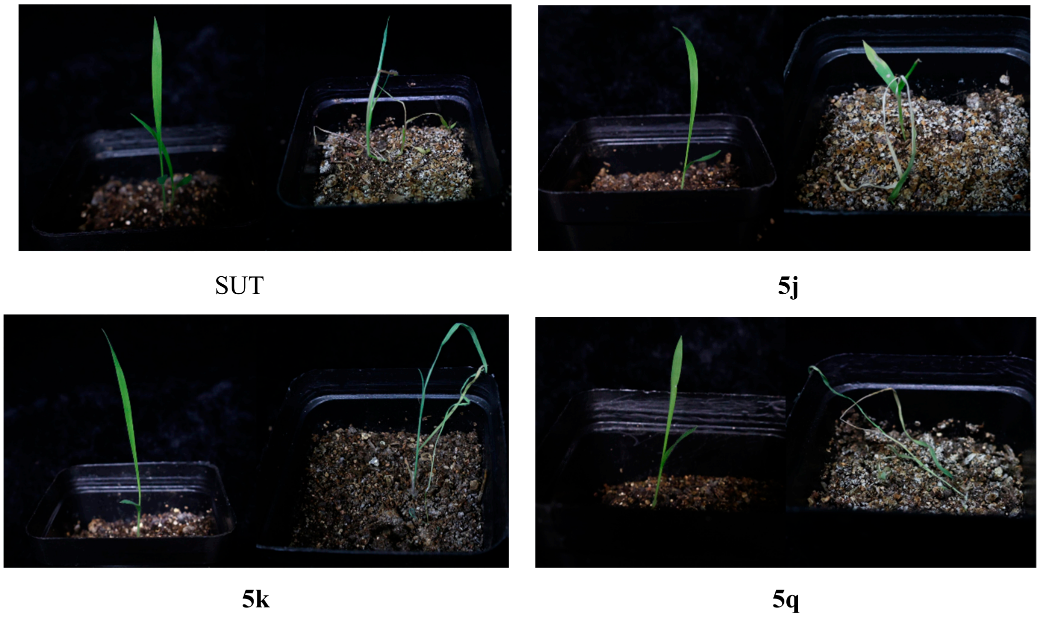

Figure S21. Postemergence herbicidal activity of Compounds 5j, 5k, 5q, SUT (150 g ai/ha, DS)

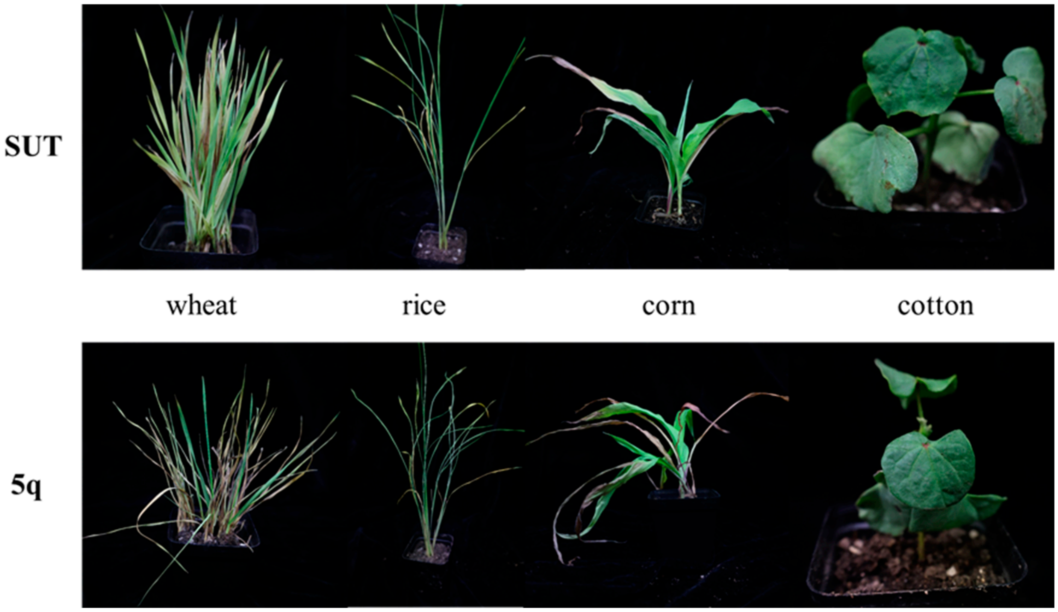

Figure S22. Crop Safety Experiment
